# Predictive and Prognostic Potential of Liver Function Assessment in Patients with Advanced Hepatocellular Carcinoma: A Systematic Literature Review

**DOI:** 10.1159/000529173

**Published:** 2023-01-17

**Authors:** Arndt Vogel, Robin K. Kelley, Philip Johnson, Philippe Merle, Thomas Yau, Masatoshi Kudo, Tim Meyer, Lorenza Rimassa

**Affiliations:** ^a^Department of Klinik für Gastroenterology, Hannover Medical School, Hannover, Germany; ^b^Helen Diller Family Comprehensive Cancer Center, University of California, San Francisco (UCSF), San Francisco, California, USA; ^c^Department of Molecular and Clinical Cancer Medicine, University of Liverpool, Liverpool, UK; ^d^Hepatology and Gastroenterology Unit, Hôpital de la Croix Rousse, Lyon, France; ^e^Queen Mary Hospital, Hong Kong, China; ^f^Department of Gastroenterology and Hepatology, Kindai University, Osaka-Sayama, Japan; ^g^Research Department of Oncology, UCL Cancer Institute, University College London, London, UK; ^h^Royal Free Hospital, London, UK; ^i^Department of Biomedical Sciences, Humanitas University, Pieve Emanuele (Milan), Italy; ^j^Medical Oncology and Hematology Unit, Humanitas Cancer Center, IRCCS Humanitas Research Hospital, Rozzano (Milan), Italy

**Keywords:** Albumin bilirubin, Child-Pugh, Advanced hepatocellular carcinoma, Liver function, Predictive potential, Prognostic potential

## Abstract

**Introduction:**

We conducted a systematic literature review to assess the utility of liver function assessments for predicting disease prognosis and response to systemic anticancer therapy in patients with advanced hepatocellular carcinoma (aHCC).

**Methods:**

This was a PRISMA-standard review and was registered with PROSPERO (CRD42021244588). MEDLINE and Embase were systematically searched (March 24, 2021) to identify publications reporting the efficacy and/or safety of systemic anticancer therapy (vs. any/no comparator) in liver-function-defined subgroups in phase 2 or 3 aHCC trials. Screening was completed by a single reviewer, with uncertainties resolved by a second reviewer and/or the authors. English-language full-text articles and congress abstracts were eligible for inclusion. Included publications were described and assessed for risk of bias using the GRADE methodology.

**Results:**

Twenty (of 2,579) screened publications were eligible; seven categorized liver function using the albumin-bilirubin system, nine using the Child-Pugh system, four using both. GRADE assessment classified ten, nine, and one publication(s) as reporting moderate-quality, low-quality, and very-low-quality evidence, respectively. Analyses of cross-trial trends of within-exposure arm analyses (active and control) reported a positive relationship between baseline liver function and overall survival and progression-free survival, supporting liver function as a prognostic marker in aHCC. There were also signals for a modest relationship between more preserved baseline liver function and extent of systemic treatment benefit, and with more preserved liver function and lower incidence of safety events.

**Conclusion:**

This review supports liver function as a prognostic variable in aHCC and highlights the value of a priori stratification of patients by baseline liver function in aHCC trials. The predictive value of liver function warrants further study. Findings were limited by the quality of available data.

## Lay Summary

Hepatocellular carcinoma (HCC) is a common type of liver cancer. Patients with advanced HCC often have some degree of liver damage that prevents their liver from functioning properly. When choosing the best treatment for patients with advanced HCC, it is important to understand the likely course of their cancer and how well they might respond to available treatment options. We carried out a review of the data from published clinical trials of advanced HCC treatments to investigate whether the quality of patients’ liver function when they start treatment predicts how their cancer will progress and how they might respond to treatment. We found that patients responded to treatment regardless of how well their liver function was working when they started therapy. However, better liver function at the start of treatment was associated with longer survival and possibly also with better treatment response. Well-designed studies are needed to explore these findings further, particularly whether liver function can help predict response to advanced HCC treatment.

## Introduction

Hepatocellular carcinoma (HCC) is a leading cause of cancer-related mortality and accounts for the majority of primary liver cancers worldwide [1, 2]. In recent years, there has been a rapid expansion of systemic, targeted therapies approved for use in patients with advanced HCC (aHCC) [3]. This increase in the number of therapeutic options available for aHCC has highlighted the importance of treatment selection and sequencing to optimize outcomes for patients. Central to informed treatment selection is understanding how key clinical characteristics may influence disease course, prognosis, and potential treatment response.

Tumour stage at diagnosis is associated with overall survival (OS) [4], and presence (and extent) of any underlying liver disease is an important clinical consideration when treating patients with HCC [5, 6, 7]. Most patients with HCC have some degree of underlying liver fibrosis (usually at the stage of cirrhosis), and the extent of liver dysfunction can have an impact on disease prognosis and treatment selection [5, 6]. Although the association between liver function and HCC prognosis is well documented, it has not been established which liver function classification system offers the greatest prognostic discrimination or association with response to systemic therapy in the context of aHCC.

In patients with HCC, liver function has traditionally been assessed by the Child-Pugh system [8], and a higher Child-Pugh score consistently correlates with shorter survival [9, 10]. Child-Pugh grading is routinely used in clinical practice to indicate the likely prognosis of patients with HCC and underlying liver disease, and a favourable grade is commonly pre-specified among HCC clinical trial inclusion criteria. Yet, the system was originally developed to assess liver function and disease prognosis in patients with liver cirrhosis and in those with portal hypertension undergoing surgery for variceal bleeding, rather than for use in patients with HCC [11, 12]. The system was developed arbitrarily and is based on (partially subjective) clinical and laboratory assessment without any formal statistical validation [7, 11, 13], with scope for variability in clinical assessment and resultant scoring [14]. In contrast, the albumin-bilirubin (ALBI) grading system is based only on serum albumin and bilirubin and consequently offers a more objective measurement of liver function compared to the Child-Pugh system. The ALBI grading system was developed empirically using data from large international databases to identify objective measures of liver function that independently influence survival in patients with HCC, thereby eliminating the dependence on subjective variables implicit within Child-Pugh assessments [7].

There are consistent reports of ALBI grading offering better discrimination than Child-Pugh grading for predicting disease prognosis in patients with HCC [15, 16, 17], but there has been less consideration of the relative utility of the two systems for predicting response to systemic HCC therapies. We conducted a systematic literature review to identify publications reporting empirical data on the association between baseline liver function and outcomes in phase 2 and 3 trials of targeted systemic aHCC therapies. Our aim was to synthesize the published literature and explore data trends indicative of baseline liver function offering predictive (as well as prognostic) potential; also of interest was whether any specific liver function grading system offered greater prognostic and/or predictive discrimination than alternative approaches.

## Materials and Methods

### Registration and Methodology

The protocol for this systematic literature review was registered with the International Prospective Register of Systematic Reviews (PROSPERO) (registration number CRD42021244588) [18]. The review was conducted in accordance with the 2020 Preferred Reporting Items for Systematic Reviews and Meta-Analyses (PRISMA) guidelines [19, 20].

### Search Strategy and Selection Criteria

Publications of interest were those reporting empirical data relating to the prognostic and/or predictive potential of liver function assessment from phase 2 or 3 clinical trials of systemic therapies for aHCC. Interventions of interest included tyrosine kinase inhibitors (TKIs), anti-vascular endothelial growth factor (VEGF) or anti-VEGF receptor (VEGFR) monoclonal antibodies (mAbs), and checkpoint inhibitors (CPIs) (see online suppl. Table. S1 at www.karger.com/doi/10.1159/000529173 for publication eligibility criteria). Eligible publications were identified by systematic searches using the Ovid bibliographic research platform, supplemented by manual handsearching of congress Websites pre-identified as being subject matter of interest. Ovid searches were run in the MEDLINE (In-Process & Other Non-Indexed Citations and Ovid MEDLINE [1946 to present]) and Embase (1974 to present) databases on March 24, 2021 (see online suppl. Table S2a, b for full search-string and screening details).

Congresses pre-identified as being of interest were those not indexed within Embase but considered by the authors to be events in which relevant data may have been reported, specifically: the European Association for the Study of the Liver (EASL) and the International Liver Cancer Association (ILCA) annual congresses 2019–2021. The congress Websites and online abstract books were manually searched to identify eligible abstracts. Relevant abstracts presented prior to January 1, 2019, were assumed to have since been published as full-text articles and identified by the Ovid searches. Abstract handsearching was carried out between March 18, 2021, and May 6, 2021. Additional to the systematic searches, relevant unpublished trial analyses known to the authors through their research networks at the time of the systematic searches were included if they were published during the data analysis phase of the review (up to October 7, 2021) and met the eligibility criteria or article inclusion.

### Publication Eligibility

Publications identified by the searches were screened against pre-specified criteria in accordance with the PRISMA 2020 guidelines [19]. The title and abstract of all identified publications were screened for eligibility against the criteria stipulated in the study protocol (summarized in online suppl. Table S1); short-listed publications underwent full-text review to confirm eligibility. Screening was completed by a single reviewer, with uncertainties resolved by a second reviewer and/or by the author group, as required.

### Data Abstraction

Standardized data were systematically extracted from eligible publications and recorded in a data extraction table (Microsoft Excel) with data fields pre-agreed by the authors. Data abstraction was conducted by two independent researchers. All relevant outcome data identified a priori in the study protocol were extracted.

### Quality and Risk of Bias Assessment

The quality and risk of bias of included publications were appraised using the Grading of Recommendations, Assessment, Development and Evaluations (GRADE) quality assessment tool. Each publication was assigned a quality rating (high, moderate, low, or very low) according to its level of potential bias, which was assessed in terms of data quality, consistency, directness, and modifying factors (precision and sparsity of data and probability of reporting bias) [21, 22]. The GRADE rating of each eligible publication is included in online Supplementary Table S3; details of the full GRADE quality appraisal are included in online Supplementary Table S4.

### Definition of Prognostic Versus Predictive Results

A methodical approach was applied to categorize extracted data according to their prognostic or predictive potential.

#### Prognostic Data

Prognostic data were defined as those reporting outcomes for within-exposure groups (either active treatment or placebo), with the only differentiating factor between the groups being baseline liver function status: for example, OS or progression-free survival (PFS) hazard ratios (HRs) for liver-function-defined subgroups ALBI 1 versus ALBI 2 and ALBI 1 versus ALBI 3 within a single exposure group. A difference in OS or PFS (shown by either HRs or absolute values) between groups stratified according to liver function was considered potentially indicative of a prognostic signal.

#### Predictive Data

Predictive data were defined as those reporting comparative treatment outcomes (investigational drug vs. placebo or vs. active comparator) in patients stratified by baseline liver function, thereby allowing a comparison of the magnitude of treatment effect in each liver-function-defined subgroup. For example, OS HR for active treatment versus placebo in the ALBI 1 subgroup was compared with OS HR for active treatment versus placebo in the ALBI 2 subgroup. A difference in the magnitude of treatment benefit between groups stratified according to liver function was considered potentially indicative of a predictive signal.

### Analysis

The prognostic and predictive potential of liver function was analysed descriptively by examination of visual and numerical trends within the data; no statistical tests were undertaken. No meta-analysis of the data was planned (or conducted) owing to expected (and confirmed) small sample sizes and data heterogeneity, including differences in population eligibility, ecology of care, liver function grading system. All relevant outcome data were analysed graphically (Fig. 2-6; online suppl. Fig. S1–4), grouped by outcome and data format; key observations are described in the Results.

### Role of the Funding Source

This systematic literature review was sponsored by Ipsen. Ipsen had no input into the design of the review, or into the analysis or interpretation of results. Ipsen sponsored the development of the manuscript in accordance with Good Publication Practice guidelines.

## Results

### Search Results

**Fig. 1.**
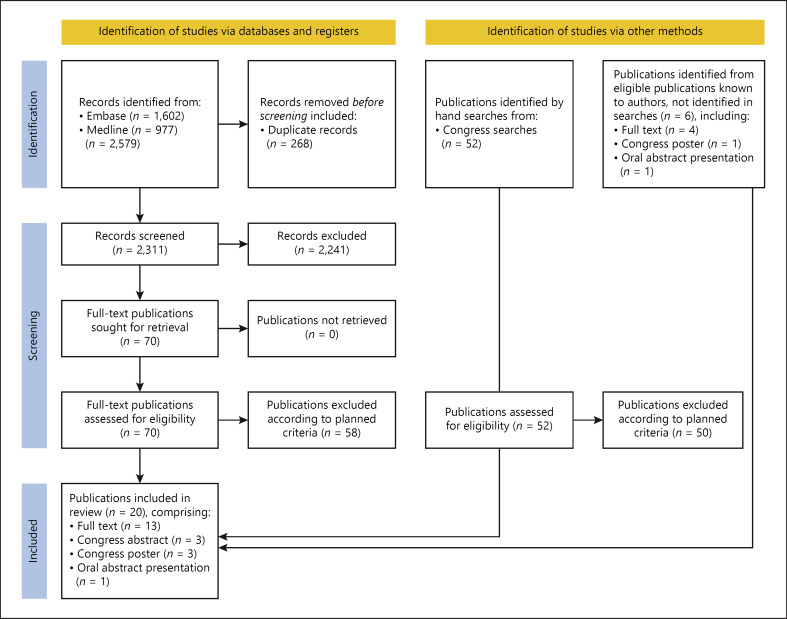
PRISMA diagram of included and excluded studies in the systematic literature review. PRISMA, Preferred Reporting Items for Systematic Reviews and Meta-Analyses.

The systematic searches identified 2,579 unique publications, of which 70 were short-listed for eligibility based on title/abstract screening and 12 were confirmed as eligible based on full-text review (eight full texts, two congress abstracts, two congress posters). Handsearching congress websites identified two additional eligible abstracts. During the data-analysis phase of the review, the authors identified a further six eligible publications (four full texts, one congress poster, one congress oral abstract). In total, 20 publications were included in the review (12 full texts, eight congress publications [three text-only abstracts, four posters, one oral presentation]). Publication identification and screening are summarized in the PRISMA flow chart (Fig. 1).

### Characteristics of Included Studies

The characteristics of the included publications are summarized in online supplementary Table S3 [7, 23, 24, 25, 26, 27, 28, 29, 30, 31, 32, 33, 34, 35, 36, 37, 38, 39, 40, 41]. Of the 20 publications included, 12 reported data from phase 3 trials, seven from phase 2 trials, and one from a phase 1/2 study. Among the eligible publications were analyses from the phase 1/2 CheckMate 040 and phase 2 Scoop-2 trials [27, 28] and the phase 3 trials BRISK-FL, SUN 1170, CELESTIAL, IMbrave150, KEYNOTE-240, REACH, REACH-2, REFLECT, and RESORCE [7, 25, 29, 33, 34, 35, 38, 39, 40].

Including studies that assessed more than one treatment modality, 18 publications reported data from trials of TKIs (included comparator arms featured: 1, brivanib; 2, cabozantinib; 2, lenvatinib; 13, sorafenib; 2, sunitinib; 1, regorafenib; 1, erlotinib), six from trials of VEGF- or VEGFR2-targeted mAbs (included comparator arms featured: 2, bevacizumab; 4, ramucirumab), and three from trials of CPIs (included comparator arms featured: 1, atezolizumab; 1, nivolumab; 1, pembrolizumab). All 20 publications reported efficacy outcomes stratified by baseline liver function [7, 23, 24, 25, 26, 27, 28, 29, 30, 31, 32, 33, 34, 35, 36, 37, 38, 39, 41], and 15 reported safety outcomes for the liver-function-defined subgroups [23, 24, 25, 26, 28, 29, 30, 31, 33, 35, 36, 38, 39, 41]. Seven publications assessed baseline liver function using the ALBI system [7, 23, 27, 33, 34, 39, 40], nine used the Child-Pugh system [24, 26, 30, 31, 32, 36, 37, 38, 41], and four used both [25, 28, 29, 35].

No eligible publications reported high-quality data according to the GRADE appraisal because of methodological limitations such as the post hoc nature of the analyses and/or the small population/subgroup sizes considered and/or (in the case of some phase 2 trials) the lack of randomization. Ten publications (50%) reported moderate-quality evidence [23, 25, 32, 33, 34, 35, 36, 37, 38, 40], indicating that further research is likely to have an important impact on confidence in the reported results, and nine (45%) low-quality evidence [7, 23, 24, 26, 28, 29, 30, 31, 39, 41], indicating that further research may change the reported effect estimate. One publication, reporting an analysis of a non-randomized phase 2 trial, offered very-low-quality evidence [27], indicating a high degree of potential uncertainty in the reported estimates (online suppl. Table S4).

### Efficacy: Prognostic Data

Descriptive analyses of the data pertaining to the prognostic potential of liver function assessments (i.e., those comparing within-trial arm outcomes, stratified by baseline liver function) were based on examination of trends within the data; no statistical tests were undertaken. In all analyses of survival estimates, the associated confidence intervals (CIs) were wide and overlapping.

#### Control (Placebo) Group Analyses

Overall, five publications reported median OS estimates for subgroups defined by baseline liver function within the placebo arms of the included trials. Examination of trends within the data consistently showed numerically shorter median OS in patients with poorer liver function at baseline (i.e., in ALBI 2 vs. ALBI 1 [29, 33, 40], Child-Pugh 6 vs. 5 [29, 38], and Child-Pugh 7 + 8 vs. 6 and vs. 5 [38]) (online suppl. Fig. S1). Across the three trials that reported median OS by ALBI grade [29, 34, 40], median OS ranged from 6.6 months to 11.4 months for ALBI 1 subgroups [29, 40] and from 4.2 months to 11.1 months for ALBI 2 subgroups [29, 34]. For the two trial analyses that reported median OS for Child-Pugh subgroups [29, 38], median OS values were 6.4 months and 9.7 months for the Child-Pugh 5 subgroups and 4.1 months and 4.8 months for the Child-Pugh 6 subgroups. In the single study that reported mean OS for patients with Child-Pugh 7/8 at baseline, median OS was 3.8 months [38]. No relationship was observed between baseline liver function and OS for patients enrolled in the control arms of the phase 3 KEYNOTE-240 trial [34] (online suppl. Fig. S1) or for the small phase 2 Scoop-2 study [27].

Only three eligible publications reported PFS for subgroups defined by baseline liver function within the relevant trials’ placebo arms [34, 38, 40] (online suppl. Fig. S2). Of these, two found no effect on PFS, while the other showed a potential relationship between poorer baseline liver function and reduced median PFS [38]: Child-Pugh 5, 2.5 months; Child-Pugh 6, 2.1 months; and Child-Pugh 7, 1.4 months.

#### Active Treatment Analyses

**Fig. 2.**
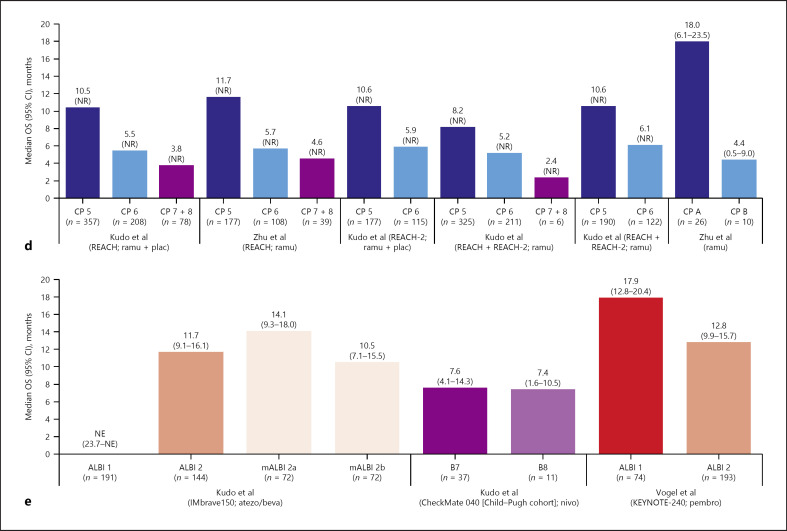
Prognostic analyses (active, or active + placebo arm): median OS estimates for trials of TKIs stratified by baseline ALBI grade (**a**) [7, 23, 33, 35, 39, 40] and Child-Pugh score (**b**) [24, 30, 31, 35, 36], for trials of VEGFR2-targeted mAbs stratified by baseline ALBI grade (**c**) [29] and Child-Pugh score (**d**) [29, 37, 38], and for trials of CPI-containing regimens (**e**) [28, 34, 39]. ALBI, albumin-bilirubin; atezo, atezolizumab; beva, bevacizumab; cabo, cabozantinib; CI, confidence interval; CP, Child-Pugh score; CPI, checkpoint inhibitor; lenva, lenvatinib; mAb, monoclonal antibody; mALBI, modified albumin-bilirubin: the modification separates ALBI 2 into subgrades 2a (>−2.60 to <−2.270) and 2b (>−2.270 to <−1.39); NE, not estimable; nivo, nivolumab; NR, not reported; OS, overall survival; pembro, pembrolizumab; plac, placebo; ramu, ramucirumab; rego, regorafenib; soraf, sorafenib; TKI, tyrosine kinase inhibitor; VEGFR2, vascular endothelial growth factor receptor 2.

In total, 15 trial publications reported median OS estimates for subgroups defined by baseline liver function within the active treatment arms. Similar to the findings reported for the placebo arms, examination of the active treatment arm data showed a consistent trend towards shorter median OS in patients with poorer baseline liver function (Fig. 2; online suppl. Fig. S3). This trend was observed for the majority of trials and broadly presented whether baseline liver function was assessed using the ALBI system (e.g., ALBI 2 vs. 1 [7, 23, 29, 33, 34, 35, 39, 40], ALBI 3 vs. 2 and vs. 1 [7, 29], and ALBI 2b vs. 2a and vs. 1 [39]) or the Child-Pugh system (e.g., Child-Pugh B vs. A [24, 30, 31, 37], Child-Pugh 6 vs. 5 [29, 35, 38], and Child-Pugh 7 + 8 vs. 6 and vs. 5 [29, 38]) (Fig. 2; online suppl Fig. S3). Across the six trials that reported median OS by ALBI grade [7, 23, 33, 35, 39, 40], median OS ranges were: ALBI 1, 8.9–17.9 months; ALBI 2, 5.0–12.8 months; and ALBI 3, 1.8–10.1 months (Fig. 2a, c, e). Among the seven trials that reported median OS stratified by Child-Pugh score [24, 29, 30, 31, 35, 36, 38], median OS ranges were Child-Pugh A, 5.5–18.0 months; Child-Pugh B, 3.2–7.4 months; Child-Pugh 5, 8.2–15.3 months; Child-Pugh 6, 5.2–9.4 months; and Child-Pugh ≥7, 2.4–7.6 months (Fig. 2b, d). The apparent trend towards shorter median OS in patients with poorer baseline liver function was consistent across trials of VEGF-targeted TKIs and mAbs, specifically trials of sorafenib [7, 23, 24, 30, 31, 35, 36, 39], cabozantinib (CELESTIAL) [40], lenvatinib (REFLECT) [35], regorafenib (RESORCE) [33] (Fig. 2a, b), and ramucirumab (a phase 2 biomarker study and REACH and REACH-2) [29, 37, 38] (Fig. 2c, d).

The trend towards shorter median OS in patients with poorer baseline liver function was also observed in the KEYNOTE-240 trial of the CPI pembrolizumab [34], in which liver function was classified by ALBI grade, and in the ALBI 2 subgroups (2b vs. 2a) in the IMbrave150 trial of atezolizumab plus bevacizumab versus sorafenib (Fig. 2e). A comparison of ALBI subgroup 2 versus 1 from the IMbrave150 trial was not possible because median OS was not evaluable for the ALBI 1 subgroup at the time of the published analysis. There was, however, no apparent difference between baseline liver function and OS for patients with Child-Pugh grade B, score 7 versus score 8, in the CheckMate 040 trial of nivolumab, although analysis was limited by the very small sample size of patients with a Child-Pugh score of 8 in this cohort (Fig. 2e) [28].

Not included in Figure 2, owing to a lack of published median OS estimates, was a phase 2 trial of sorafenib versus bevacizumab plus erlotinib [32]. Examination of the Kaplan-Meier graphs in the trial publication, however, suggested a consistent relationship between poorer baseline liver function and shorter OS because median OS was approximately 12 months for patients with a Child-Pugh score of A compared with approximately 6 months for those with Child-Pugh B7.

In a post hoc analysis of the REFLECT trial of lenvatinib versus sorafenib, median OS was shorter in patients whose liver function deteriorated from Child-Pugh grade A to B (vs. remained as grade A) over the first 8 weeks after randomization [26]. In the lenvatinib arm, median (95% CI) OS was 6.8 (2.6–10.3) months in patients whose liver function deteriorated from Child-Pugh A to B over the 8-week period (*n* = 60) compared with 13.3 (11.6–16.1) months for patients who still had Child-Pugh grade A at week 8 (*n* = 413). Similarly, median (95% CI) OS was 4.5 (2.9–6.1) months in the subgroup of patients in the sorafenib arm whose liver function had deteriorated from Child-Pugh A to B (*n* = 47) by week 8 compared with 12.0 (10.2–14.0) months for those who retained Child-Pugh grade A status throughout the 8-week period [26]. A similar analysis was conducted for the CELESTIAL trial population, but no efficacy (only safety) outcomes were reported for liver-function-defined subgroups [41]. These post hoc analyses are not included in Figure 2 owing to the difference in their analysis approach, which assessed the relationship between rate of liver function decline (rather than extent of dysfunction at baseline) and patient outcomes.

**Fig. 3.**
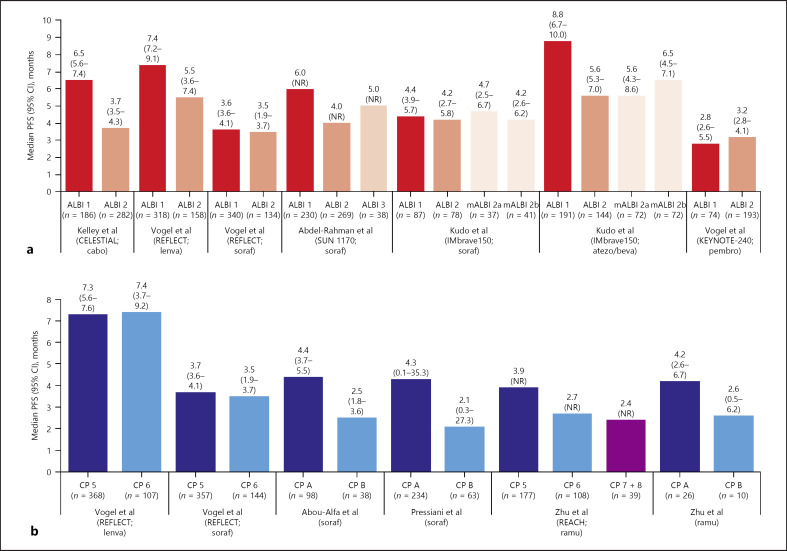
Prognostic data (active treatment arm): median PFS estimates stratified by liver-function-defined subgroups according to baseline ALBI grade (**a**) [23, 34, 35, 39, 40] and Child-Pugh score (**b**) [24, 30, 35, 37, 38]. ALBI, albumin-bilirubin grade; atezo, atezolizumab; beva, bevacizumab; cabo, cabozantinib; CI, confidence interval; CP, Child-Pugh score; lenva, lenvatinib; mALBI, modified albumin-bilirubin: the modification separates ALBI 2 into subgrades 2a (>−2.60 to <−2.270) and 2b (>−2.270 to <−1.39). NR = not reported; pembro, pembrolizumab; PFS, progression-free survival; ramu, ramucirumab; soraf, sorafenib.

The relationship between baseline liver function and PFS was evaluated for the active treatment arms of nine of the eligible publications (Fig. 3). As for median OS, there was a possible trend towards shorter median PFS in patients with poorer baseline liver function, but the signal was weaker than for OS (Fig. 3 vs. Fig. 2, respectively). Across the six trials that reported median PFS by ALBI grade [23, 34, 35, 39, 40], median PFS across subgroups ranged from 2.8 months to 8.8 months for the ALBI 1 subgroups and from 3.2 months to 5.6 months for the ALBI 2 subgroups (Fig. 3a). Across the trials that reported median PFS by Child-Pugh score, median PFS ranges were Child-Pugh A, 4.2–4.4 months; Child-Pugh B, 2.1–2.6 months; Child-Pugh 5, 3.7–7.3 months; and 2.7–7.4 months for Child-Pugh 6 (Fig. 3b).

The signal for shorter median PFS in patients with poorer baseline liver function was apparent for the cabozantinib arms of the CELESTIAL trial [40] and for the lenvatinib arm of the REFLECT trial [35] but was not evident for the sorafenib arm of REFLECT (Fig. 3a). Considering sorafenib specifically, there was also no apparent relationship between the baseline liver function and median PFS duration in the sorafenib arm of the phase 3 IMbrave150 trial [39], but there was a possible signal in the sorafenib arm of the phase 3 SUN 1170 trial [23] (Fig. 3a) and a stronger signal in two phase 2 trials by Abou-Alfa et al. [24] and Pressiani et al. [30] (Fig. 3b).

A trend towards shorter median PFS in patients with poorer baseline liver function was also visible in the REACH and REACH-2 trials of ramucirumab [37, 38] and in the combination atezolizumab plus bevacizumab arm of the IMbrave150 trial [39] (Fig. 3a). No trend between poorer baseline liver function and shorter median PFS was evident in the KEYNOTE-240 trial of pembrolizumab [34] (Fig. 3a).

### Efficacy: Predictive Data

Data pertaining to the predictive potential of liver function assessments (i.e., those reporting survival outcomes for the experimental treatment vs. control arm, stratified by baseline liver function) were available from seven trials (five trials with placebo control arms [25, 29, 33, 34, 40] and two with active control arms [35, 39]). Predictive analyses were based on descriptive assessment of potential trends between HR for the experimental versus control arms within each individual trial; no statistical tests were undertaken. In all seven trials, the CIs around the HRs were wide and largely overlapping.

**Fig. 4.**
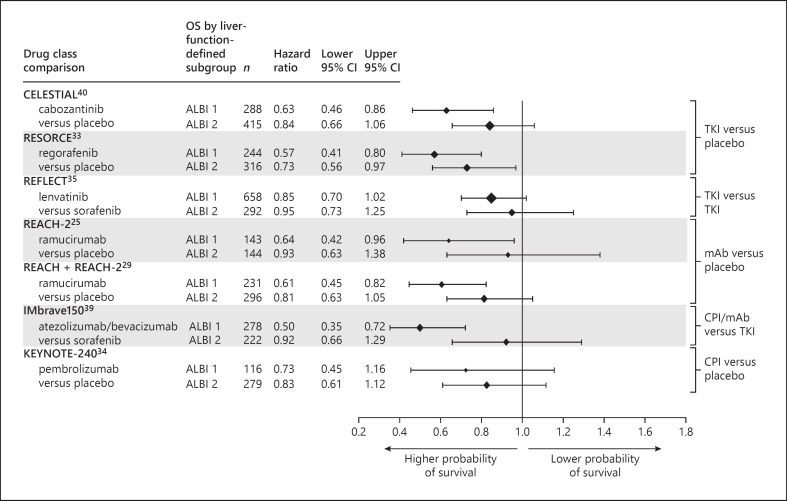
Predictive data: OS forest plot for between-treatment-arm analyses (investigational drug vs. comparator), stratified by baseline liver function [25, 29, 33, 34, 35, 39, 40]. ALBI, albumin-bilirubin grade; CI, confidence interval; CPI, checkpoint inhibitor; mAb, monoclonal antibody; OS, overall survival; TKI, tyrosine kinase inhibitor.

**Fig. 5.**
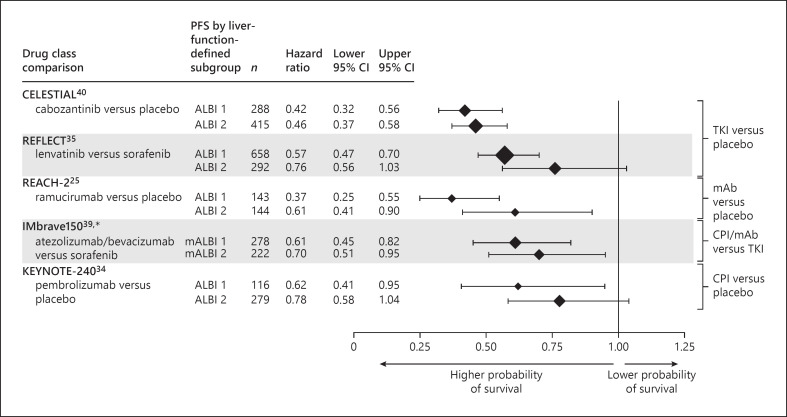
Predictive analysis: PFS forest plot for between-treatment-arm analyses (active vs. placebo), stratified by liver function [25, 34, 35, 39, 40]. ALBI, albumin-bilirubin grade; CI, confidence interval; CPI, checkpoint inhibitor; mAb, monoclonal antibody; PFS, progression-free survival; TKI, tyrosine kinase inhibitor.

Overall, patients with poorer liver function at baseline demonstrated slightly higher HRs (lesser treatment benefit) compared with those for patients with more preserved liver function (OS, Fig. 4; PFS, Fig. 5). The within-trial separation of HRs for the different liver function subgroups was more evident in trials that stratified patients according to baseline ALBI (OS, Fig. 4; PFS, Fig. 5) rather than Child-Pugh grade (online suppl. Fig. S4), although there were limited comparative data available. Across the studies that reported HR for OS for experimental treatment versus the trial comparator arm stratified by ALBI grade [25, 29, 33, 34, 35, 39, 40] and Child-Pugh score [25, 35], OS HRs ranges were ALBI 1, 0.61–0.73 (placebo comparator) and 0.50–0.85 (sorafenib comparator); ALBI 2, 0.73–0.93 (placebo comparator) and 0.92–0.95 (sorafenib comparator) (Fig. 4); Child-Pugh 5, 0.70 (placebo comparator) and 0.91 (sorafenib comparator); Child-Pugh 6, 0.82 (placebo comparator) and 0.91 (sorafenib comparator) (online suppl. Fig. S4a). Across the studies that reported PFS HRs for the experimental treatment versus the comparator arm, stratified by ALBI grade [29, 34, 35, 39, 40] and Child-Pugh score [25, 35], HRs for PFS ranges were ALBI 1, 0.37–0.62 (placebo comparator) and 0.57–0.61 (sorafenib comparator); ALBI 2, 0.46–0.78 (placebo comparator) and 0.70–0.76 (sorafenib comparator) (Fig. 5); Child-Pugh 5, 0.43 (placebo comparator) and 0.63 (sorafenib comparator); Child-Pugh 6, 0.56 (placebo comparator) and 0.65 (sorafenib comparator) (online suppl. Fig. S4b).

Despite the modest signal for greater treatment benefit in patients with more preserved liver function, the evidence to support liver function as a predictor of systemic treatment response was less strong than for its role as a prognostic variable. In the predictive analyses, the directional benefit of the HRs for the preserved liver function and liver dysfunction subgroups was consistent and mirrored that for the overall populations, both for the endpoint of death (OS, Fig. 4) and for disease progression (PFS, Fig. 5). Taken together, these data suggest that patients with more preserved liver function may derive modestly greater benefit from systemic therapy, but overall, baseline liver function did not appear to have substantial predictive value.

#### Overall Survival

Consistent, yet modest, differences in OS HRs for subgroups defined by baseline ALBI grade were seen in the data from the CELESTIAL trial of cabozantinib [40], the RESORCE trial of regorafenib [33], the REACH-2 trial (and pooled analysis of the REACH/REACH-2 trials) of ramucirumab [25, 29], the IMbrave150 trial of atezolizumab plus bevacizumab versus sorafenib [39], and from the KEYNOTE-240 trial of pembrolizumab [34] (Fig. 4). Two publications, Vogel et al. and Brandi et al. [25, 35], evaluated the differences in OS HRs according to degree of liver dysfunction using both the ALBI and Child-Pugh systems, providing cursory insight into the relative potential of the two systems to predict OS. In both cases, there was a suggestion that ALBI may provide better predictive discrimination than Child-Pugh categorization, but there was wide uncertainty in the data (Fig. 4; online suppl. Fig. S4a).

#### Progression-Free Survival

Modestly higher HRs for patients with less (vs. more) preserved liver function were seen in the PFS analyses of five trials: the CELESTIAL trial of cabozantinib [40], the REFLECT trial of lenvatinib versus sorafenib [35], the REACH-2 trial of ramucirumab [25, 29], the IMbrave150 trial of atezolizumab plus bevacizumab versus sorafenib [39], and the KEYNOTE-240 trial of pembrolizumab [34] (Fig. 5). In the two publications that evaluated PFS for subgroups defined according to the ALBI and Child-Pugh classification systems (Vogel et al. and Brandi et al. [25, 35]), similar to the OS analyses, any modest separation of the HRs according to degree of liver dysfunction was more evident in the ALBI-based analyses (Fig. 5; online suppl. Fig. S4b).

### Safety

**Fig. 6.**
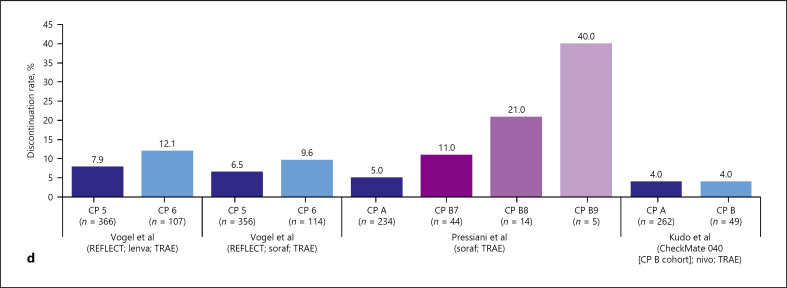
Safety data (active treatment arm): rates of grade ≥3 AEs stratified by ALBI grade (**a**) [33, 35, 39, 40] and Child-Pugh score/grade (**b**) [28, 31, 35, 38] and discontinuation rates stratified by baseline ALBI grade (**c**) [29, 33, 35, 39, 40] and Child-Pugh score/grade (**d**) [28, 30, 35]. AE, adverse event; ALBI, albumin-bilirubin grade; atezo, atezolizumab; beva, bevacizumab; cabo, cabozantinib; CP, Child-Pugh score; lenva, lenvatinib; mALBI, modified albumin-bilirubin: the modification separates ALBI 2 into subgrades 2a (>−2.60 to <−2.270) and 2b (>−2.270 to <−1.39); nivo, nivolumab; ramu, ramucirumab; rego, regorafenib; soraf, sorafenib; TEAE, treatment-emergent adverse event; TRAE, treatment-related adverse event.

As illustrated by the results of the prognostic and predictive analyses, patients with more preserved liver function are more likely to have longer time on treatment and, thus, longer time to accrue treatment-emergent and treatment-related adverse events (TEAEs and TRAEs). Relative rates of safety outcomes for different baseline liver function subgroups within the active treatment arms of the included publications were therefore described to explore the potential for liver function assessment to predict on-treatment safety signals, considering particular safety events adjudicated as being treatment related by trial clinicians and/or independent review boards. Overall, 13 eligible trials reported safety outcomes for different baseline liver function subgroups. The safety data for the active treatment arms of the included trial publications are summarized in Tables 1 and 2 and Figures 6 and in online supplementary Table S5 for the placebo arms.

In trials involving active investigational TKI arms, there was a consistent trend for higher rates of TEAEs and/or TRAEs in patients with poorer baseline liver function [28, 31, 33, 35, 38, 39] (Fig. 6a, b). The exception was the CELESTIAL trial, in which rates were lower in the ALBI 2 (vs. 1) group, but these were any adverse events (AEs) rather than TRAEs [40].

The same trend was true for the VEGFR2-targeted mAb ramucirumab, but data were only available from one trial [38]. There was no clear signal in the two trials involving CPIs, either as monotherapy [28] (Fig. 6b) or combination therapy [39] (Fig. 6a).

More evident than a relationship between AEs and baseline liver function in the included publications was a potential relationship between treatment discontinuation and baseline liver function (Fig. 6c, d). Rates of treatment discontinuation (whether recorded as treatment related or any, depending on the source publication) were consistently higher in patients with poorer baseline liver function status. This potential signal was consistent across all investigational TKI [30, 33, 35, 39, 40], mAb [29, 39], and CPI regimens, with the exception of the CheckMate 040 evaluation of nivolumab, in which no difference was seen in discontinuation rates related to TRAEs, potentially owing to lack of power [28].

## Conclusions

This systematic literature review was designed to identify and describe data trends in clinical trials of systemic anticancer therapies in patients with aHCC that reported outcomes stratified by patients’ baseline liver function. The aim was to explore the potential trends in the data indicative of a role for baseline liver function assessments in predicting not only the disease course of patients with aHCC but also their response to therapy.

Twenty publications were identified that reported trial outcomes stratified by baseline liver function. All 20 publications reported efficacy outcomes; 13 also reported safety outcomes.

Comparisons of efficacy outcomes between subgroups defined by baseline liver function corroborated previous assertions of liver function as a prognostic factor for patients with HCC receiving systemic treatment [5, 6]. In both the control and active treatment arms of the included trials, there were consistent trends for shorter survival in patients with greater liver dysfunction at baseline. The signal was stronger for OS than for PFS, which is not unexpected, given that OS estimates can be influenced by post-progression therapy and that survival may be extended by the use of effective post-progression treatment approaches in patients who maintain good liver function after initial systemic therapy. It is important to note that patients may have several reasons for a deterioration in liver function, including progression of underlying liver cirrhosis, treatment-related toxicity, and/or tumour progression. For an individual patient, the exact cause of their liver function deterioration may be multifaceted and challenging to establish. It should also be noted that the risk of death resulting from the natural history of cirrhosis is a potential confounder of PFS outcomes in HCC trials; time to progression may be a better surrogate endpoint for evaluating the benefits of effective drugs [1].

Of greater novelty in this review was the suggestion that liver function assessments may have modest predictive value with respect to systemic treatment response. In a number of the included trials of efficacious aHCC systemic therapies, there was separation of the HRs (for OS and PFS) for different baseline ALBI subgroups, favouring patients with more preserved liver function. However, the HRs consistently indicated treatment benefit (vs. respective comparators) across preserved liver function and liver dysfunction subgroups and across endpoints. These findings mirrored those for the overall populations and suggest that liver function has limited value as a predictive biomarker. Thus, overall, despite a possible signal for greater treatment benefit in patients with more preserved liver function, the data did not suggest substantial predictive utility of liver assessment for predicting differential treatment response; the direction of OS and PFS HRs (vs. comparator therapies) was consistent across the preserved liver function, the liver dysfunction subgroups, and the overall populations. There were limited data to inform a comparison of the relative discriminatory ability of the ALBI and Child-Pugh systems, but two publications provided a possible signal for ALBI being more sensitive than Child-Pugh in discerning the modest potential predictive utility of baseline liver function [25, 33].

Despite a lack of consistency in AE reporting across the included trials (some reported any AEs, others TEAEs or TRAEs), among patients receiving active treatment, there was generally a trend for higher AE rates in those with greater baseline liver dysfunction. More pronounced than the signals in the AE data was the apparent relationship between severity of liver dysfunction and rates of treatment discontinuation among patients receiving active therapy. By contrast, data from the large, observational GIDEON study of sorafenib suggested that AE incidence was consistent across Child-Pugh subgroups and reported no substantial differences in discontinuations resulting from drug-related AEs in the real-world setting [42].

The prognostic utility of liver function assessments described is in general agreement with the existing literature and with the clinical understanding that patients with better liver function are often able to tolerate more lines of treatment and can experience prolonged survival. This finding, together with the novel suggestion of a possible modest signal for greater (and/or prolonged) benefit of systemic therapy in patents with more preserved liver function at the time of treatment initiation, underscores the clinical relevance of liver function assessments for informing clinical practice. These findings emphasize the importance of close monitoring and management of co-existing liver disease in patients receiving local and systemic treatment and of exploring the reason behind liver function deterioration (be it underlying cirrhosis, tumour progression, and/or treatment toxicity). Additionally, they highlight the value of trying to preserve (or improve) liver function, where feasible, and the relevance of considering liver function when deciding whether to transition patients from local to systemic therapy. An algorithm has recently been proposed that uses liver functional reserve as the starting point for a more systematic approach to decision-making around non-surgical HCC treatments [43].

Within the research context, this review confirms the utility of liver function as a stratification factor in clinical trials of patients with aHCC. Adequately powered analyses of liver-function-defined subgroups could help to refine the optimum assessment approach and allow corroboration (or not) of the potential predictive utility of liver function assessments reported here. In addition, the review suggests value in conducting dedicated safety and efficacy studies in patients with greater extents of hepatic dysfunction due to the potential impact of liver function on treatment response as well as prognosis.

A limitation of the review was its reliance on data from aHCC trials involving populations with relatively preserved liver function, a reflection of the convention for trials of investigational aHCC therapies to restricted recruitment to Child-Pugh A liver disease and to exclude patients with significant liver dysfunction. Regardless, the eligible trials did permit some degree of population stratification by extent of liver function, but the associated outcome analyses were frequently post hoc and generally involved underpowered subgroups. As a result, although only publications reporting data from phase 2 and 3 trials were eligible for inclusion, the majority of papers provided moderate-, low-, or very-low-grade evidence. These quality ratings reflect a number of limitations and areas of potential bias in the source data, such as small sample sizes, lack of randomization, the post hoc nature of many of the analyses, and/or the pooling of data across multiple trials involving populations with differing baseline characteristics. Indeed, all survival estimates had wide-associated CIs, which were largely overlapping between subgroups. Some trials did not support a relationship between liver function and disease or treatment outcomes, which may be due, in part, to more favourable safety profiles of individual agents in patients with greater liver dysfunction and to potential differences in the extent of liver function chronicity and irreversibility between the populations of the included trials.

Another limitation of the review is the heterogeneity of the trials included (involving different interventions, control arms, and patient populations), as well as differences in liver function assessment approaches (both at the system and individual-clinician level). For these reasons, direct interpretation of the data should focus on within-trial analyses, and descriptive trends in the data should be considered as hypothesis generating only. No formal statistical analyses were undertaken or were possible.

In conclusion, this review corroborates liver function as a valuable prognostic variable in patients with aHCC and suggests additional, modest utility for predicting extent of benefit from systemic therapy for aHCC. Overall, the observations support the inclusion of liver function assessments with discriminatory potential among the a priori stratification factors used in future HCC trials.

## Statement of Ethics

This systematic literature review did not require ethical approval or written informed consent in accordance with local/national guidelines as we used only publicly available data.

## Conflict of Interest Statement

Arndt Vogel: speaker, consultancy, and advisory fees from AstraZeneca, Bayer, BMS, BTG, Daiichi-Sankyo, EISAI, GSK, Imaging Equipment Ltd (AAA), Incyte, Ipsen, Lilly, Merck, MSD, Novartis, PierreFabre, Roche, Sanofi, Servier, Sirtex, and Terumo. R. Katie Kelley: consulting fees (to institution) from Agios, AstraZeneca, BMS, and Merck; consulting fees (to self) from Exact Sciences, Genentech/Roche, Gilead, and Kinnate; travel support to satellite symposium from Ipsen; and research support (to institution) from Agios, AstraZeneca, Bayer, BMS, Eli Lilly, EMD Serono, Exelixis, Genentech/Roche, LOXO oncology, Merck, Novartis, Partner Therapeutics, Relay Therapeutics, Surface Oncology, and Taiho. Philip Johnson: no conflicts of interest to disclose. Philippe Merle: advisory board fees from AstraZeneca, Bayer, Eisai, Lilly, Merck, and Roche and grants and advisory board fees from Genosciences and Ipsen. Thomas Yau: consulting or advisory fees and honoraria from AbbVie, AstraZeneca, Bayer, BMS, Eisai, Eli Lilly, EMD Serono, Exelixis, H3 Biomedicine, Ipsen, Merck Sharp & Dohme Corp., Novartis, New B Innovation, OrigiMed, Pfizer, SillaJen, Sirtex, and Taiho. Masatoshi Kudo: honoraria and consulting or advisory fees from Bayer AG and Eisai Co. Ltd.; honoraria from Bayer AG, Bristol Myers Squibb, EA Pharma, Eisai, and Merck Sharp & Dohme; and research funding from Bayer, Chugai Pharmaceutical, Daiichi-Sankyo, Merck Sharp & Dohme, Ono Pharmaceutical, Otsuka Pharmaceutical, Sumitomo Dainippon Pharma, and Taiho Pharmaceutical. Tim Meyer: consulting fees from Adaptimmune, AstraZeneca, BMS, Boston Scientific, Eisai, Ipsen, and Roche. Lorenza Rimassai: consulting fees from Amgen, ArQule, AstraZeneca, Basilea, Bayer, BMS, Celgene, Eisai, Exelixis, Genenta, Hengrui, Incyte, Ipsen, IQVIA, Lilly, MSD, Nerviano Medical Sciences, Roche, Sanofi, Servier, Taiho Oncology, and Zymeworks; lecture fees from AbbVie, Amgen, Bayer, Eisai, Gilead, Incyte, Ipsen, Lilly, Merck Serono, Roche, and Sanofi; travel expenses from Ipsen; and institutional research funding from Agios, ARMO BioSciences, AstraZeneca, BeiGene, Eisai, Exelixis, Fibrogen, Incyte, Ipsen, Lilly, MSD, Nerviano Medical Sciences, Roche, and Zymeworks.

## Funding Sources

This study was funded by Ipsen. TM is supported by the NIHR UCLH Biomedical Research Centre.

## Author Contributions

Arndt Vogel contributed to the conception of the systematic literature review, design of the review, interpretation of the results, drafting of the paper, or to revising it critically for intellectual content and gave final approval of the version to be published and agreed to be accountable for all aspects of the work. Robin K. Kelley, Philip Johnson, Philippe Merle, Thomas Yau, Masatoshi Kudo, Tim Meyer, and Lorenza Rimassai contributed to the design of the review, interpretation of the results, drafting of the paper, or to revising it critically for intellectual content and gave final approval of the version to be published and agreed to be accountable for all aspects of the work.

## Data Availability Statement

Qualified researchers may request access to patient-level study data that underlie the results reported in this publication. Additional relevant study documents, including the clinical study report, study protocol with any amendments, annotated case report form, statistical analysis plan, and dataset specifications, may also be made available. Patient-level data will be anonymized, and study documents will be redacted to protect the privacy of study participants.

Where applicable, data from eligible studies are available 6 months after the studied medicine and indication have been approved in the USA and EU or after the primary manuscript describing the results has been accepted for publication, whichever is later. Further details on Ipsen’s sharing criteria, eligible studies, and process for sharing are available here (https://vivli.org/members/ourmembers/). Any requests should be submitted to www.vivli.org for assessment by an independent scientific review board.

## Supplementary Material

Supplementary data

## Figures and Tables

**Table 1:** Safety outcomes of patients receiving active treatment, stratified by liver function subgroups defined by ALBI grade

First author Study name Study identifier	Intervention (drug class) *N*	Liver function safety subgroups, *n*	Grade ≥3 AEs, %	Discontinuation rate, %	Dose reductions due to AEs, %	Signal for potential relationship between baseline liver function and safety outcomes
Kelley et al. [40] CELESTIAL NCT01908426	Cabozantinib (TKI) *N =* 707 (cabozantinib: *n =* 470; placebo: *n =* 237)	ALBI 1:186 ALBI 2:279	**ALBI 1 versus ALBI 2** Any: 75% versus 63% PPE: 18% versus 16% Hypertension: 22% versus 12% AST increased: 8% versus 14% Fatigue: 8% versus 13% Diarrhoea: 13% versus 8% Asthenia: 4% versus 9% Decreased appetite: 5% versus 6% Anaemia: 3% versus 5%	**ALBI 1 versus ALBI 2** 12% versus 19%	NR	**Yes** ♦ AEs: rate of AEs in ALBI 1 and ALBI 2 subgroups consistent with the overall population ♦ Treatment-related discontinuation: higher in ALBI 2 versus ALBI 1

Vogel et al. [33] RESORCE NCT01774344	Regorafenib (TKI) *N =* 573 (regorafenib: *n =* 379; placebo: *n =* 194)	ALBI 1:163 ALBI 2:209	**ALBI 1 versus ALBI 2** Any: 76% versus 82% Hypertension: 20% versus 11% HFSR: 17% versus 9% Serious TEAE: 34% versus 53% **TEAEs** Grade 3:59% versus 53% Grade 4: 7% versus 14% Grade 5:10% versus 15% **TRAEs** Grade 3:47% versus 46% Grade 4:3% versus 4% Grade 5:1% versus 2%	**ALBI 1 versus ALBI 2, due to the following** TEAEs: 18% versus 29% TRAEs: 7% versus 13%	**ALBI 1 versus ALBI 2, due to the following** TEAEs: 65% versus 71% TRAEs: 50% versus 58%	**Possibly** ♦ Grade 3/4 TEAEs were similar between ALBI grades ♦ ALBI grade 1 (vs. ALBI grade 2) was associated with a lower rate of serious TEAEs and lower rates of treatment discontinuation due to TEAEs and TRAEs

Abdel-Rahman et al. [23] SUN 1170 NCT00699374	Sorafenib (TKI) *N =* 544	ALBI 1:230 ALBI 2:269 ALBI 3:38 ALBI unknown/ missing: 7	NR	NR	NR	**Yes** Grade ≥3 AEs, OR (95% Cl) versus ALBI 1 ♦ ALBI 2:1.628 (1.065–2.487) (*p* = 0.024) ♦ ALBI 3:0.804 (0.381–1.696) (*p* = 0.566)

Vogel et al. [35]* REFLECT NCT01761266	Sorafenib (TKI) versus lenvatinib (TKI) *N =* 954 (lenvatinib: *n =* 478; sorafenib: *n =* 476)	ALBI 1:658 ALBI 2:292	**ALBI 1 versus ALBI 2 (lenvatinib only)** 69.5% versus 86.1 %	**ALBI 1 versus ALBI 2 (lenvatinib only)** 6.6% versus 13.3%	**ALBI 1 versus ALBI 2 (lenvatinib only)** 35.5% versus 39.9%	**Yes** Patients receiving lenvatinib with ALBI 2 had higher rates (vs. ALBI 1) of the following ♦ grade ≥3 TEAEs ♦ study-drug withdrawal/discontinuation Rates of dose reduction were similar

Kudo et al. [29] REACH and REACH-2 NCT01140347 NCT02435433	Ramucirumab (mAb) *N = 8* 57 (REACH: *N =* 565 [ramucirumab, *n =* 283; placebo, *n =* 282]; REACH-2: *N =* 292 [ramucirumab, *n =* 197; placebo, *n =* 95])	ALBI 1:136 ALBI 2:176	NR Grade >3 liver injury for ramucirumab ALBI 1 versus ALBI 2 11.8% versus 26.1%	**Overall discontinuation** **rate** ALBI 1 versus ALBI 2 13.2% versus 18.2%	NR	**Yes** ♦ Patients with a higher ALBI grade at baseline had increased incidence of AESIs in both the ramucirumab and placebo arms

Brandi et al. [25]* REACH-2 NCT02435433	Ramucirumab (mAb) *N* = 292	Ramucirumab **+** placebo ALBI 1:143 ALBI 2:144 ALBI missing: 5	NR	NR	NR	**Yes** ♦ ALBI 2 (vs. ALBI 1) associated with a higher proportion of grade ≥3 TEAEs

Kudo et al. [39] IMbrave150 NCT03434379	Atezolizumab (CPI) + bevacizumab (mAb) *N* = 501 (atezolizumab + bevacizumab: *n =* 336; sorafenib: *n =* 165)	ALBI 1:270 mALBI 2a: 108 mALBI 2b: 107	**TRAEs; ALBI 1 versus ALBI 2a versus ALBI 2b** Atezolizumab **+** bevacizumab 48% versus 38% versus 38% Sorafenib 46% versus 57% versus 37%	**ALBI 1 versus ALBI 2a versus ALBI 2b, due to TRAEs** Atezolizumab **+** bevacizumab 17% versus 25% versus 30% Sorafenib 9% versus 14% versus 16%	**ALBI 1 versus ALBI 2a versus ALBI 2b, due to TRAEs** Atezolizumab **+** bevacizumab: none permitted Sorafenib 37% versus 38% versus 37%	**Possibly** ♦Patients with a higher ALBI grade at baseline had increased incidence of discontinuing therapy due to TRAEs ♦There was no clear association between baseline ALBI classification and grade ≥3 TRAEs or (sorafenib) dose reductions

AE, adverse event; AESI, adverse event of special interest; ALBI, albumin-bilirubin grade; AST, aspartate aminotransferase; Cl, confidence interval; CPI, checkpoint inhibitor; HFSR, hand-foot skin reaction; mAb, monoclonal antibody; mALBI, modified albumin-bilirubin; NCT, National Clinical Trial; NR, not reported; OR, odds ratio; PPE, palmar-plantar erythrodysesthesia; TEAE, treatment-emergent adverse event; TKI, tyrosine kinase inhibitor; TRAE, treatment-related adverse event. *Stratified results according to ALBI grade and by Child-Pugh score.

**Table 2:** Safety outcomes of patients receiving active treatment, stratified by liver function subgroups defined by Child-Pugh score

First author Study name Study identifier	Intervention (drug class) *N*	Liver function safety subgroups, *n*	Grade ≥3 AEs, %	Discontinuation rate, %	Dose reductions due to AEs, %	Signal for potential relationship between baseline liver function and safety outcomes
El-Khoueiry et al. [41]* CELESTIAL NCT01908426	Cabozantinib (TKI) *N = 70J*	Cabozantinib* ♦ CP B (51) **Placebo** ♦ CP B (21)	**All-causality grade 3/4 AEs** Overall population: 68% CP B: 71% **Most common all-causality grade 3/4 AEs; overall population versus cabozantinib-treated CP B patients** Fatigue: 10% versus 20% Ascites: 4% versus 14% AST increase: 12% versus 14% Thrombocytopenia: 3% versus 12% PPE: 17% versus 8% Hypertension: 16% versus 8%	**Discontinuation due to TRAEs** Overall population: 16% CP B: 18%	Overall population: 62% CP B: 61%	**No** Tolerability of cabozantinib in CP B subgroup was similar to the overall study population, although rates of specific AEs differed between the two groups

Huynh et al. [26]^†^ REFLECT NCT01761266	Lenvatinib (TKI) *N =* 473	**Lenvatinib** CP A:413 CP B: 60 **Sorafenib** CP A: 427 CP B: 47	**TRAEs per patient-years** **CP A versus CP B** Lenvatinib: 1.4% versus 3.7% Sorafenib: 1.7% versus 3.4%	**TRAEs leading to withdrawal (rate per patient-year)** **CP A versus CP B** Lenvatinib: 0.12% versus 0.58% Sorafenib: 0.16% versus 0.40%	**TRAEs leading to dose reduction (rate per patient-year)** **CP A versus CP B** Lenvatinib: 0.76% versus 1.77% Sorafenib: 0.94% versus 1.61%	**Yes** ♦ Signal for higher rates of grade ≥3 TRAEs; related dose reductions and treatment discontinuations in CP B versus CP A

Vogel et al. [35]^‡^ REFLECT NCT01761266	Sorafenib (TKI) versus lenvatinib (TKI) *N =* 1,675 (954 patients enrolled in REFLECT, lenvatinib: *n =* 478; sorafenib: *n =* 476)	**Pooled** CP 5: 722 CP 6: 221	CP 5 **versus CP 6 (lenvatinib only)** 71.6% versus 86.0%	**CP 5 versus CP 6 (lenvatinib only)** 7.9% versus 12.1%	**CP 5 versus CP 6 (lenvatinib only)** 36.6% versus 39.3%	**Yes** Patients receiving lenvatinib with CP 6 had higher rates (vs. CP 5) of the following ♦ grade ≥3 TRAEs ♦ study-drug withdrawal/ discontinuation Rates of dose reduction were similar

Abou-Alfa et al. [24] NA (phase 2 study)	Sorafenib (TKI) *N* = 137	CP A: 98 CP B: 38 CP missing: 1	**Most common TRAEs; CP A versus CP B Grade 3** Diarrhoea: 8% versus 8% HFSR: 5% versus 5% Fatigue: 4% versus 8% **Grade 3–4** Bilirubin: 14% versus 53% Ascites: 3% versus 5% Encephalopathy: 3% versus 13% **Grade 4 TRAEs** No grade 4 toxicities were observed	NR	**CP A versus CP B** 31% versus 21%	**Unclear** ♦ Higher frequency of some AEs but lower rates of dose reductions in CP B versus CP A group

Suzuki et al. [31] UMIN Clinical Trials Registry 000002972	Sorafenib (TKI) *N* **=** 52	CP A: 40 CP B: 12	**CP A versus CP B** Any: 77.5% versus 91.6% Thrombocytopenia: 10% versus 25% HFSR: 27.5% versus 16.7% Erythema multiforme: 0% versus 16.7% Upper Gl bleeding: 0% versus 16.7% Treatment-related deaths: 0% versus 0%	**Reason for discontinuation;** **CP A versus CP B** Radiologically confirmed progressive disease: 90% versus 58.3% Clinically confirmed tumour progression: 5% versus 8.3% Unacceptable toxicities: 2.5% versus 16.7% Patient request: 2.5% versus 16.7%	NR	Yes ♦ Grade 3 or 4 TRAEs were consistently higher in the CP B (vs. CP A) subgroup

Yau et al. [36] NA (phase 2 study)	Sorafenib (TKI) *N* = 51	CP A: 36 CP B: 13 CP C: 2	**CP A versus CP B or C (p value)** Haematologic toxicities: 17% versus 33% (0.18) Non-haematologic toxicities: 47% versus 47% (0.97) Liver function derangement: 56% versus 73% (0.24)	NR	NR	**No** ♦ No significant differences between CP subgroups in terms of grade 3 or 4 haematologic toxicities, non-haematologic toxicities, or liver function derangement

Pressiani et al. [30] NA	Sorafenib (TKI) *N =* 297	CP A: 234 CP B7:44 CP B8:14 CP B9:5	**CP A versus CP B (p value)** Cachexia: 6% versus 10% (0.043) Fatigue: 16% versus 11% (0.351) Weight loss: 4% versus 0% (0.212) HFSR: 12% versus 5% (0.109) Rash: 5% versus 0% (0.077) Diarrhoea: 15% versus 13% (0.711) Abdominal pain: 5% versus 2% (0.313) Liver failure: 3% versus 5% (0.031) Anaemia: 6% versus 2% (0.315)	**CP A versus B7 versus B8 versus B9** Overall: 12% versus 43% versus 57% versus 40% Due to AEs: 5% versus 11% versus 21% versus 40%	**CP A versus CP B** 37% versus 21%	**Unclear** **♦**Statistically significantly higher rates of only some grade ≥3 AEs in patients with CP B versus A **♦**Rates of treatment discontinuation increased with greater extent of baseline liver dysfunction **♦**Rates of dose reductions lower in patients with more severe baseline liver dysfunction

Zhu et al. [38] REACH NCT01140347	Ramucirumab (mAb) *N =* 644 (ramucirumab: *n* = 324; placebo: *n =* 319)	CP 5:173 CP 6:106 CP 7 or 8:38	**CP 5 versus CP 6 versus CP 7 or 8** 59•0% versus 64•2% versus 92•1% **Grade ≥3** Peripheral oedema: 0.0% versus 0.9% versus 0.0% Fatigue: 1.7% versus 3.8% versus 5.3% Headache: 1.2% versus 0.0% versus 0.0% Hypertension: 16.2% versus 5.7% versus 2.6% Decreased appetite: 1.7% versus 2.8% versus 5.3% Abdominal pain: 2.9% versus 0.0% versus 5.3% Ascites: 3.5% versus 6.6% versus 15.8% Asthenia: 58% versus 3.8% versus 10.5%	NR	NR	**Yes** ♦ Patients with CP 7 or 8 (vs. CP 6 or CP 5) had higher rates of the following ♦ Overall grade ≥3 TEAEs, including higher rates of fatigue, decreased appetite, ascites ♦ AESIs, including higher rates of liver injury and/or failure and of bleeding and/or haemorrhage

Brandi et al. [25]^‡^ REACH-2 NCT02435433 Kudo et al. [28] CheckMate 040 – Child-Pugh B cohort NCT01658878	Ramucirumab (mAb) *N = 292* Nivolumab (CPI) *N* = 49	**Ramucirumab** + **placebo** CP 5: 177 CP 6: 115 **Nivolumab** CP A: 262 CP B: 49 (patients with CP A were from trial cohorts 1 and 2 and were included in for comparison only)	**AESI grade ≥3** Liver injury and/or failure: 17.3% versus 27.4% versus 57.9% Bleeding: 6.4% versus 4.7% versus 10.5% Hypertension: 16.2% versus 6.6% versus 2.6% Infusion-related reactions: 0.6% versus 1.9% versus 2.6% NR **Grade** 3 **or** 4 **TRAE: CP A versus** B; n (%) Any: 23% versus 24% Pruritus: 0.4% versus 0% Amylase increased: 3% versus 4% AST increased: 6% versus 4% Lipase increased: 6% versus 2% ALT increased: 4% versus 0% LFT increased: 0.4% versus 0% Hypertransaminasemia: 0% versus 4% Hepatic function abnormal: NR versus 2% Hepatitis: 5% versus 2% Rash: 1% versus 2%	NR **CP A versus** B;n (%) Overall: 95% versus 96% Due to drug toxicity: 6% versus 4%	NR NR 4%	**Yes** ♦CP 6 (vs. CP 5) associated with a higher proportion of grade ≥3 TEAEs **Yes** ♦Incidence of TRAEs higher for CP A versus CP B ♦Incidence of grade 3/4 TRAEs or similar

AE, adverse event; AESI, adverse event of special interest; ALBI, albumin-bilirubin grade; ALT, alanine aminotransferase. AST, aspartate aminotransferase. CP, Child-Pugh score. CPI, checkpoint inhibitor; Gl, gastrointestinal; HFSR, hand-foot skin reaction; LFT, liver function test; mAb, monoclonal antibody; NCT, National Clinical Trial; NR, not reported; PPE, palmar-plantar erythrodysesthesia; TEAE, treatment-emergent adverse event; TKI, tyrosine kinase inhibitor; TRAE, treatment-related adverse vent; UMIN, University hospital Medical Information Network.

* Retrospectively analysed data from patients in CELESTIAL whose cirrhosis evolved to Child-Pugh B by week 8 versus overall population (eligible patients had baseline CP A liver function).

† Post hoc exploratory analysis of key efficacy and safety outcomes in patients from REFLECT whose liver function had deteriorated to CP B versus those whose liver function remained CP A in the 8 weeks after randomization.

‡ Stratified results according to ALBI grade and by Child-Pugh score.
