# Supplementary material for: Predictive and Prognostic Potential of Liver Function Assessment in Patients with Advanced Hepatocellular Carcinoma: A Systematic Literature Review
Source: Liver Cancer. 2023 Jan 17;12(4):372–91. doi: 10.1159/000529173 (PMC10561324; doi:10.1159/000529173)
Supplement: Supplementary file 1 — Supplementary data [file lic-0012-0372-s01.docx]

**Supplementary materials
Predictive and prognostic potential of liver function assessment in patients with advanced hepatocellular carcinoma: a systematic literature review**

Arndt Vogel^a^, R. Katie Kelley^b^, Philip Johnson^c^, Philippe Merle^d^, Thomas Yau^e^, Masatoshi Kudo^f^, Tim Meyer^g,h^, Lorenza Rimassa^i,j^

^a^Department of Klinik für Gastroenterology, Hannover Medical School, Hannover, Germany;

^b^Helen Diller Family Comprehensive Cancer Center, University of California, San Francisco (UCSF), San Francisco, CA, USA;

^c^Department of Molecular and Clinical Cancer Medicine, University of Liverpool, Liverpool, UK;

^d^Hepatology and Gastroenterology Unit, Hôpital de la Croix Rousse, Lyon, France;

^e^Queen Mary Hospital, Hong Kong, China;

^f^Department of Gastroenterology and Hepatology, Kindai University, Osaka-Sayama, Japan;

^g^Research Department of Oncology, UCL Cancer Institute, University College London, UK;

^h^Royal Free Hospital, London, UK;

^i^Department of Biomedical Sciences, Humanitas University, Pieve Emanuele (Milan), Italy;

^j^Medical Oncology and Hematology Unit, Humanitas Cancer Center, IRCCS Humanitas Research Hospital, Rozzano (Milan), Italy

Short Title: Liver function assessment in patients with aHCC

Correspondence to: Professor Arndt Vogel,

Department of Gastroenterology, Hepatology and Endokrinology,

Hannover Medical School

Carl-Neuberg Str. 1, 30625,

Hannover, Germany.

T: +495115329590

E: [**vogel.ardnt@mh-hannover.de**](mailto:vogel.ardnt@mh-hannover.de)

| **Category** | **Inclusion criteria*** |
| --- | --- |
| Population | - Patients with aHCC |
| Interventions | - Apatinib, atezolizumab,avelumab, axitinib, bevacizumab, brivanib, cabozantinib, camrelizumab, cediranib, dovitinib, durvalumab, erlotinib, everolimus, ipilimumab, lenvatinib, linifanib, nivolumab, pembrolizumab, ramucirumab, regorafenib, S-1, sorafenib, sunitinib, tivantinib, and tremelimumab |
| Comparator | - Placebo, active treatments - No comparator |
| Outcomes | - Clinical outcomes (ORR, OS, PFS, DCR, TTP, other survival and response outcomes) - AEs^†^ |
| Study designs | - Phase 2 or 3 clinical trials - Evidence for patient stratification by liver function |
| Date restrictions | - Full-text publications not restricted by date - Congress publications published between January 2019 and during the data-analysis phase of the systematic review (September 2021^‡^) |
| Country restrictions | - No restriction |
| Language | - English language |

***Supplementary Table S1.*** **Eligibility criteria for studies identified by systematic literature review***Systematic reviews, editorials, narrative reviews, MAs/NMAs, modelling studies, and case studies/series were excluded.

^†^AEs including number of treatment dose reductions and discontinuations associated with AEs.

^‡^Extended from PROSPERO date to ensure capture and inclusion of most up-to-date data.

AE=adverse event. aHCC=advanced hepatocellular carcinoma. DCR=disease control rate. MA=meta-analysis. NMA=network meta-analysis. ORR=objective response rate. OS=overall survival. PFS=progression-free survival. PROSPERO= International Prospective Register of Systematic Reviews. TT=time to progression.

| **Number** | **Searches** | **Hits** |
| --- | --- | --- |
| 1 | liver cell carcinoma/ | 166 638 |
| 2 | (Hepatocellular carcinoma or hepatocellular cancer or HCC or liver carcinoma or liver cancer or liver cell carcinoma or hepatic cell carcinoma or hepatic carcinoma or hepatic cancer or hepatocarcinoma or hepatoma).ti,ab. | 202 108 |
| 3 | 1 or 2 | 233 334 |
| 4 | (sorafenib or lenvatinib or regorafenib or cabozantinib or ramucirumab or atezolizumab or bevacizumab or pembrolizumab or nivolumab or ipilimumab or sunitinib or brivanib or cediranib or linifanib or dovitinib or camrelizumab or apatinib or tivantinib or durvalumab or tremelimumab or everolimus, or erlotinib or avelumab or axitinib or S-1).ti,ab. | 230 862 |
| 5 | sorafenib/ or lenvatinib/ or regorafenib/ or cabozantinib/ or ramucirumab/ or atezolizumab/ or bevacizumab/ or pembrolizumab/ or nivolumab/ or ipilimumab/ or sunitinib/ or brivanib/ or cediranib/ or linifanib/ or dovitinib/ or camrelizumab/ or apatinib/ or tivantinib/ or durvalumab/ or tremelimumab/ or everolimus/ or erlotinib/ or avelumab/ or axitinib/ or S-1/ | 176 288 |
| 6 | 4 or 5 | 304 167 |
| 7 | 3 and 6 | 16 808 |
| 8 | Clinical Trial/ | 1 008 368 |
| 9 | Randomized Controlled Trial/ | 654 550 |
| 10 | controlled clinical trial/ | 467 383 |
| 11 | multicenter study/ | 284 568 |
| 12 | Phase 3 clinical trial/ | 52 699 |
| 13 | Phase 4 clinical trial/ | 4277 |
| 14 | exp RANDOMIZATION/ | 91 109 |
| 15 | Single Blind Procedure/ | 42 512 |
| 16 | Double Blind Procedure/ | 183 330 |
| 17 | Crossover Procedure/ | 66 674 |
| 18 | PLACEBO/ | 366 294 |
| 19 | randomi?ed controlled trial$.tw. | 254 620 |
| 20 | rct.tw. | 41 483 |
| 21 | (random$ adj2 allocat$).tw. | 46 327 |
| 22 | single blind$.tw. | 26 801 |
| 23 | double blind$.tw. | 219 663 |
| 24 | ((treble or triple) adj blind$).tw. | 1325 |
| 25 | placebo$.tw. | 325 120 |
| 26 | Prospective Study/ | 675 265 |
| 27 | or/8-26 | 2 489 731 |
| 28 | Case Study/ | 77 588 |
| 29 | case report.tw. | 446 150 |
| 30 | abstract report/ or letter/ | 1 194 819 |
| 31 | Editorial.pt. | 690 287 |
| 32 | Letter.pt. | 1 171 597 |
| 33 | Note.pt. | 848 213 |
| 34 | or/28-33 | 3 313 522 |
| 35 | 27 not 34 | 2 361 169 |
| 36 | (single arm or phase 1 or phase I).ti,ab. | 120 833 |
| 37 | 35 not 36 | 2 311 113 |
| 38 | 7 and 37 | 3555 |
| 39 | "review"/ | 2 593 166 |
| 40 | conference abstract.pt. | 4 066 914 |
| 41 | 39 or 40 | 6 660 080 |
| 42 | 38 not 41 | 1238 |
| 43 | 38 and 40 | 1398 |
| 44 | limit 43 to yr="2019 - 2021" | 456 |
| 45 | 42 or 44 | 1694 |
| 46 | remove duplicates from 45 | 1602 |

***Supplementary Table S2a.*** **Search terms run in Embase (March 24, 2021)**

| **Number** | **Searches** | **Hits** |
| --- | --- | --- |
| 1 | exp liver neoplasms/ | 169880 |
| 2 | (Hepatocellular carcinoma or hepatocellular cancer or HCC or liver carcinoma or liver cancer or liver cell carcinoma or hepatic cell carcinoma or hepatic carcinoma or hepatic cancer or hepatocarcinoma or hepatoma).ti,ab. | 139039 |
| 3 | 1 or 2 | 221118 |
| 4 | sorafenib.mp. | 9494 |
| 5 | lenvatinib.mp. | 943 |
| 6 | regorafenib.mp. | 1324 |
| 7 | cabozantinib.mp. | 1024 |
| 8 | ramucirumab.mp. | 911 |
| 9 | atezolizumab.mp. | 1488 |
| 10 | bevacizumab.mp. | 19117 |
| 11 | pembrolizumab.mp. | 5202 |
| 12 | nivolumab.mp. | 6256 |
| 13 | ipilimumab.mp. | 4082 |
| 14 | sunitinib.mp. | 6488 |
| 15 | brivanib.mp. | 130 |
| 16 | cediranib.mp. | 391 |
| 17 | linifanib.mp. | 75 |
| 18 | dovitinib.mp. | 147 |
| 19 | camrelizumab.mp. | 92 |
| 20 | apatinib.mp. | 663 |
| 21 | tivantinib.mp. | 150 |
| 22 | durvalumab.mp. | 743 |
| 23 | tremelimumab.mp. | 330 |
| 24 | everolimus.mp. | 7772 |
| 25 | erlotinib.mp. | 7095 |
| 26 | avelumab.mp. | 565 |
| 27 | axitinib.mp. | 1113 |
| 28 | S-1.mp. | 60047 |
| 29 | or/4-28 | 119925 |
| 30 | Randomized Controlled Trials as Topic/ | 141673 |
| 31 | randomized controlled trial/ | 525722 |
| 32 | Random Allocation/ | 104926 |
| 33 | Double Blind Method/ | 163113 |
| 34 | Single Blind Method/ | 29905 |
| 35 | clinical trial/ | 527985 |
| 36 | clinical trial, phase i.pt. | 21402 |
| 37 | clinical trial, phase ii.pt. | 34415 |
| 38 | clinical trial, phase iii.pt. | 18124 |
| 39 | clinical trial, phase iv.pt. | 2073 |
| 40 | controlled clinical trial.pt. | 94101 |
| 41 | randomized controlled trial.pt. | 525722 |
| 42 | multicenter study.pt. | 290633 |
| 43 | clinical trial.pt. | 527985 |
| 44 | exp Clinical Trials as topic/ | 354266 |
| 45 | or/30-44 | 1415885 |
| 46 | (clinical adj trial$).tw. | 392902 |
| 47 | ((singl$ or doubl$ or treb$ or tripl$) adj (blind$3 or mask$3)).tw. | 178778 |
| 48 | PLACEBOS/ | 35401 |
| 49 | placebo$.tw. | 223378 |
| 50 | randomly allocated.tw. | 30613 |
| 51 | (allocated adj2 random$).tw. | 34018 |
| 52 | or/46-51 | 670756 |
| 53 | 45 or 52 | 1703581 |
| 54 | case report.tw. | 330240 |
| 55 | letter/ | 1128183 |
| 56 | historical article/ | 362667 |
| 57 | or/54-56 | 1804439 |
| 58 | 53 not 57 | 1664778 |
| 59 | (single arm or phase 1 or phase I).ti,ab. | 68115 |
| 60 | 58 not 59 | 1625669 |
| 61 | 3 and 29 and 60 | 1386 |
| 62 | "review"/ | 2773954 |
| 63 | 61 not 62 | 977 |

***Supplementary Table S2b***. **Search terms run in MEDLINE(R) and Epub Ahead of Print, In-Process, In-Data-Review & Other Non-Indexed Citations, Daily and Versions(R) 1946 to March 23, 2021 (March 24, 2021)**

| **First author name Publication year**  **Publication type** | **Country** | **Trial phase (NCT)** | **Trial design** | **Intervention \| comparator** | **Treatment dose** | **Outcomes** | **N (total patients in trial)** | **Liver-function-defined subgroups (n)** | **GRADE evidence rating** |
| --- | --- | --- | --- | --- | --- | --- | --- | --- | --- |
| **Publications using ALBI system only** | | | | | | | | | |
| Abdel‑Rahman et al 2018 [1]  Full-text article | Multinational | SUN 1170  Phase 3  (NCT00699374) | Randomised | Sunitinib \| sorafenib | Sunitinib malate 37·5 mg OD or sorafenib 400 mg BID | Primary: OS  Other: PFS, safety | 1074 | **Sorafenib**  • ALBI 1 (230)  • ALBI 2 (269)  • ALBI 3 (38)  • Unknown grade (7) | Moderate |
| Johnson et al 2015 [2]  Full-text article | Multinational | Phase 3[3, 4]  NCT00699374  [SUN 1170]  NCT00858871 [BRISK-FL] | Randomised (pooled analysis of individual patient data from 2 RCTs) | NCT00699374 [SUN 1170]  sunitinib \| sorafenib  NCT00858871 [BRISK-FL]):  brivanib \| sorafenib | NCT00699374:  sunitinib 37·5 mg OD  Sorafenib 400 mg BID  NCT00858871 [BRISK-FL]):  brivanib 800 mg OD  Sorafenib 400 mg BID | Primary: OS | 1132 | • ALBI 1 (475)  • ALBI 2 (542)  • ALBI 3 (11) | Low |
| Kelley et al 2021 [5]  Full-text article | Multinational | CELESTIAL  Phase 3 (NCT01908426) | Randomised, double-blind | Cabozantinib \| placebo | 60 mg OD | Primary: OS  Other: PFS, ORR, safety | 707 | **Cabozantinib**  • ALBI 1 (186)  • ALBI 2 (282)  • ALBI 3 (2)  **Placebo**  • ALBI 1 (102)  • ALBI 2 (133)  • ALBI 3 (2) | Moderate |
| Kobayashi et al 2020 [6]*  Congress abstract | Japan | Scoop-II trial  Phase 2 | Randomised | HAIC with cisplatin followed by sorafenib \| Sorafenib | Sorafenib 400 mg BID or HAIC with cisplatin followed by sorafenib 400 mg BID | Primary: Not specified  All endpoints: OS, TTP | 68 | **Sorafenib**  • mALBI 1–2a (16) • mALBI 2b (17)  **HAIC**  • mALBI 1–2a (24)  • mALBI 2b (11) | Very low |
| Kudo et al 2021 [7]*  Oral abstract presentation | Multinational | IMbrave150  Phase 3  (NCT03434379) | Randomised | Atezolizumab and bevacizumab \| sorafenib | Atezolizumab 1200 mg Q3W + bevacizumab 15 mg/kg Q3W or  sorafenib 400 mg BID | Primary: OS, PFS  Other: Time to deterioration of liver function, safety | 501 | **Atezolizumab + bevacizumab**  • ALBI 1 (191)  • mALBI 2a (72)  • mALBI 2b (72)• Unknown grade (1)  **Sorafenib**  • ALBI 1 (87)  • mALBI 2a (37)  • mALBI 2b (41) | Low |
| Vogel et al 2020 [8]  Congress poster | Multinational | KEYNOTE-240  Phase 3  (NCT02702401) | Randomised, double-blind | Pembrolizumab and BSC \| placebo and BSC | 200 mg Q3W | Primary: OS, PFS | 413 | **Pembrolizumab**  • ALBI 1 (74)  • ALBI 2 (193)  **Placebo**  • ALBI 1 (42)  • ALBI 2 (86) | Moderate |
| Vogel et al 2021 [9]  Congress poster | Multinational | RESORCE  Phase 3  (NCT01774344)[10] | Randomised, double-blind | Regorafenib \| placebo | Regorafenib 160 mg OD for weeks 1–3 of each 4-week cycle | Primary: OS  Other: Safety | 573 | **Regorafenib**  • ALBI 1 (164)  • ALBI 2 (213)  • ALBI 3 (1)  • Grade missing (1)  **Placebo**  • ALBI 1 (81)  • ALBI 2 (112)  • ALBI 3 (1) | Moderate |
| **Publications using Child–Pugh system only** | | | | | | | | | |
| Abou-Alfa et al 2011 [11]  Full-text article | Multinational | Phase 2 | Single arm | Sorafenib \| none | 400 mg BID | Primary: tumour response  Other: PFS, TTP, OS, safety | 137 | • CP A (98)  • CP B (38)  • CP status ‘missing’ (1) | Low |
| El-Khoueiry et al 2020 [12]^†^  Congress abstract | Multinational | CELESTIAL  Phase 3 (NCT01908426) | Randomised, double-blind | Cabozantinib \| placebo | 60 mg OD | Primary: OS  Other: PFS, ORR, safety | 707 | **Cabozantinib**  • CP B (51)  **Placebo**  • CP B (22) | Low |
| Huynh et al 2021 [13]^‡^  Congress poster | Multinational | REFLECT  Phase 3  (NCT01761266) [14] | Randomised | Lenvatinib \| sorafenib | Lenvatinib (per BW:  12 mg OD for ≥60 kg; 8 mg OD for <60 kg) or sorafenib (400 mg BID) | Primary: OS  Other: PFS, ORR, safety | 947 | **Lenvatinib^†^**  • CP A (413)  • CP B (60)  **Sorafenib**  • CP A (427)  • CP B (47) | Low |
| Pressiani et al 2013 [15]  Full-text article | Italy | Phase 2 | Part 1: Single arm  Part 2: Randomised, (entered on radiological progression) | Part 1: sorafenib \| none  Part 2: sorafenib \| BSC | Part 1: 400 mg BID  Part 2: 600 mg BID | Primary: Not specified  All endpoints: OS, PFS, TTP,  safety | 297 | **Part 1**  • CP A (234)  • CP B7 (44)  • CP B8 (14)  • CP B9 (5) | Low |
| Suzuki et al 2018 [16]  Full-text article | Japan | Phase 2  (UMIN Clinical Trials Registry 000002972) | Single arm | Sorafenib \| none | 400 mg BID | Primary: TTP  Other: OS, ORR, tumour response, safety | 52 | • CP A (40)  • CP B (12) | Low |
| Thomas et al 2018 [17]  Full-text article | USA | Phase 2  (NCT00881751) | Randomised | Bevacizumab and erlotinib \| sorafenib | 10 mg/kg bevacizumab every 14 days and 150 mg erlotinib OD or 400 mg sorafenib BID | Primary: OS | 95 | **Sorafenib**  • CP A (38)  • CP B7 (5)  **Bevacizumab**  • CP A (47)  • CP B7 (8) | Moderate |
| Yau et al 2009 [18]  Full-text article | Hong Kong | Phase 2 | Single arm | Sorafenib \|none | 400 mg BID | Primary: OS  Other: Safety | 51 | • CP A (36)  • CP B (13)  • CP C (2) | Moderate |
| Zhu et al 2013 [19]  Full-text article | Multinational | Phase 2 | Single arm | Ramucirumab \| none | 8 mg/kg Q2W | Primary: PFS  Other: OS | 43 | • CP A (26)  • CP B (10) | Moderate |
| Zhu et al 2017 [20]  Full-text article | Multinational | REACH  Phase 3  (NCT01140347) | Randomised, double-blind | Ramucirumab \| placebo | 8 mg/kg Q2W | Primary: OS  Other: PFS, ORR, DCR, safety | 644 | **Pooled ramucirumab/placebo**  • CP 5 (357)  • CP 6 (208)  • CP 7 and 8 (78)  **Ramucirumab**  • CP 5 (177)  • CP 6 (108)  • CP 7 and 8 (39)  **Placebo**  • CP 5 (180)  • CP 6 (100)  • CP 7 and 8 (39) | Moderate |
| **Publications using ALBI and Child–Pugh systems** | | | | | | | | | |
| Brandi et al 2019 [21]  Congress abstract | Multinational | REACH-2  Phase 3  (NCT02435433) [22] | Randomised, double-blind | Ramucirumab \| placebo | 8 mg/kg Q2W | Primary: OS  Other: PFS, safety | 292 | **Pooled treatment arms**  **ALBI**  • ALBI 1 (143)  • ALBI 2 (144)  • Missing grade (5)  **Child–Pugh**  • CP 5 (177)  • CP 6 (115) | Moderate |
| Kudo et al 2021 [23]  Full-text article | Multinational | REACH and REACH-2  Phase 3  (NCT01140347 and NCT02435433) | Randomised, double-blind | Ramucirumab (R) \| placebo (P) | 8 mg/kg Q2W | Primary: OS  Other: Safety | 857 | **Pooled AFP ≥400 ng/mL and CP A**  **ALBI** • ALBI 1: 231 (R=136; P=95)  • ALBI 2: 296 (R=176; P=120)  • ALBI 3: 7 (R=1; P=6)  **Child–Pugh**  • CP 5: 325 (R=190; P=135)  • CP 6: 211 (R=122; P=89)  • CP 7 and 8: 6 (R=4; P=2) | Low |
| Kudo et al 2021 [24]  Full-text article | Multinational | CheckMate 040 (Child–Pugh B cohort)  Phase 1/2  (NCT01658878) | Single arm | Nivolumab | 240 mg Q2W | Primary: ORR, duration of response  Other: OS, DCR, safety | 49 | **ALBI**  • ALBI 2 (44)  • ALBI 3 (5)  **Child–Pugh**  • CP 6 (1)  • CP 7 (37)  • CP 8 (11) | Low |
| Vogel et al 2021 [25]  Full-text article | Multinational | REFLECT  Phase 3  (NCT01761266) | Randomised | Lenvatinib \| sorafenib | Lenvatinib 8 mg (BW <60 kg) or 12 mg (BW ≥60 kg) OD or sorafenib 400 mg BID | Primary: OS  Other: PFS, ORR, safety | 954 | **ALBI**  • ALBI 1 (lenva [318]; soraf [340])  • ALBI 2 (lenva [158]; soraf [134])  **Child–Pugh**  • CP 5 (lenva [368]; soraf [357])  • CP 6 (lenva [107]; soraf [114]) | Moderate |

***Supplementary Table S3.* Study characteristics and quality assessment of included studies**

*Modified ALBI grading was used in study (ALBI grade 2 was divided into ALBI 2a and 2b) [26].

^†^Retrospectively analysed data from patients in CELESTIAL whose cirrhosis evolved to Child–Pugh B by week 8 versus overall population (eligible patients had baseline CP A liver function).

^‡^*Post* *hoc* exploratory analysis of key efficacy and safety outcomes in patients from REFLECT whose liver function had deteriorated to CP B versus those whose liver function remained CP A in the 8 weeks after randomisation.

AFP=alpha-fetoprotein. ALBI=albumin–bilirubin grade. BID=twice daily. BSC=best supportive care. BW=body weight. CP=Child–Pugh score. DCR=disease control rate. GRADE=Grading of Recommendations, Assessment, Development and Evaluations. HAIC=hepatic arterial infusion chemotherapy. lenva=lenvatinib. mALBI=modified albumin–bilirubin. NCT=National Clinical Trial. OD=once daily. ORR=objective response rate. OS=overall survival. PFS=progression-free survival. Q2W=every 2 weeks. Q3W=every 3 weeks. RCT=randomised controlled trial. soraf=sorafenib. TTP=time to progression. UMIN=University hospital Medical Information Network.

| **Author** | **Publication type** | **Design** | **Quality** | **Quality limitation/ comment** | **Consistency** | **Consistency limitation/ comment** | **Directness** | **Directness** | **Modifying factors** | **Modifying limitation/ comment** | **Quality (default for RCTs = high)** |
| --- | --- | --- | --- | --- | --- | --- | --- | --- | --- | --- | --- |
| Abdel-Rahman et al 2018 [1]  SUN 1170 | Full-text | Randomised trial = high | –1: serious limitation to study quality | Open-label  'secondary' analysis of RCT  Population largely Asian (76%) | No important inconsistency | NA | No uncertainty about directness | NA | –1: imprecise or sparse data | Small patient sizes Potential for confounding due to open-label design | Moderate |
| Abou-Alfa et al 2011 [11] | Full-text | Non-randomised trial = moderate | –1: serious limitation to study quality | Single-arm, non-randomised trial  Retrospective analysis  Population mostly CP A patients | –1: serious limitation to study quality | Study population was made up of different tumour grades including HCC | No uncertainty about directness | NA | –1: imprecise or sparse data | Small patient sizes, not powered for significance | Low |
| Brandi et al 2019 [21]  REACH-2 | Abstract | Randomised trial = high | –1: serious limitation to study quality | Retrospective analysis conducted according to ALBI and CP status | No important inconsistency | NA | No uncertainty about directness | NA | –1: imprecise or sparse data | Small patient sizes, not powered for significance | Moderate |
| El-Khoueiry et al 2020 [12]  CELESTIAL | Abstract | Randomised trial = high | –1: serious limitation to study quality | Retrospective analysis of patients who were CP B at the end of week 8 | –1: important inconsistency concerns | Baseline characteristics differed between treatment groups, specifically macrovascular invasion, extrahepatic spread, elevated alpha-fetoprotein, HBV, and HCV. Patients with ALBI 1, 2, and 3 also differed between treatment groups | No uncertainty about directness | NA | –1: imprecise or sparse data | Small patient sizes, not powered for significance | Low |
| Huynh et al 2021 [13]  REFLECT | Poster | Randomised trial = high | –1: serious limitation to study quality | Retrospective analysis of CP A and B patients  Data descriptive and not analysed for significance | –1: important inconsistency concerns | Baseline characteristics differ between CP groups | No uncertainty about directness | NA | –1: imprecise or sparse data | Small patient sample size for CP A patients, not powered for significance | Low |
| Johnson et al 2015 [2] | Full-text | Randomised trial = high | –1: serious limitation to study quality | Retrospective analysis conducted according to ALBI and CP status | –1: important inconsistency concerns | Baseline characteristics differed by trial centre location. CP scores not available (only grades) to directly compare to ALBI scores | No uncertainty about directness | NA | –1: high probability of reporting bias | Potential for confounding due to differences in baseline characteristics between groups | Low |
| Kobayashi et al 2020 [6]*  Scoop-II | Abstract | Randomised trial = high | –1: serious limitation to study quality | Sub-analysis of patients with impaired liver function | –1: important inconsistency concerns | It is implied but not shown that baseline characteristics, including differences in liver function, may account for differences in response | No uncertainty about directness | NA | –1: imprecise or sparse data | Small patient sample size | Very low |
| Kudo et al 2021 [23]  REACH+REACH-2 | Full-text | Randomised trial = high | –1: serious limitation to study quality | Pooled data from two different studies  Exploratory analysis | No important inconsistency | NA | No uncertainty about directness | NA | –1: high probability of reporting bias | Subgroup analysis (ie, not powered for significance) | Low |
| Kudo et al 2021 [24]  CheckMate 040 | Full-text | Non-randomised trial = moderate | –1: serious limitation to study quality | Open-label, single-arm study  Population largely CP B | –1: important inconsistency concerns | Population consists of naïve and previously treated patients | –1: some uncertainty about directness | Indirect comparisons made | –1: imprecise or sparse data | Small patient size not powered for significance | Low |
| Kudo et al 2021 [7]*  IMbrave150 | Presentation | Randomised trial = high | –1: serious limitation to study quality | Retrospective exploratory analysis of IMbrave150 trial | –1: important inconsistency concerns | Imbalances between mALBI groups make comparisons difficult to interpret | No uncertainty about directness | NA | –1: imprecise or sparse data | Small number of patients in each cohort and not powered for significance | Low |
| Kelley et al 2021 [5]  CELESTIAL | Full-text | Randomised trial = high | –1: serious limitation to study quality | Retrospective analysis of CELESTIAL trial | –1: important inconsistency concerns | Comparisons of ALBI 1 and 2 data are limited by the differences in baseline characteristics of these two populations | No uncertainty about directness | NA | No modifying factors | NA | Moderate |
| Pressiani et al 2013 [15] | Full-text | Non-randomised trial = moderate | –1: serious limitation to study quality | Open-label, single-arm study comparing CP A and B patients | –1: important inconsistency concerns | Differences in CP status impact patient/treatment outcomes | No uncertainty about directness | NA | –1: imprecise or sparse data | Almost 80% of patients are in the CP A group (n=234), CP B (n=63) | Low |
| Suzuki et al 2018 [16] | Full-text | Non-randomised trial = moderate | –1: serious limitation to study quality | Single-arm study comparing CP A and B patients | –1: important inconsistency concerns | Differences in CP status impact patient/treatment outcomes | No uncertainty about directness | NA | –1: imprecise or sparse data | 77% of patients were CP A (n=40), remainder were CP B (n=12), and only some CP B patients were eligible for sorafenib | Low |
| Thomas et al 2018 [17] | Full-text | Randomised trial = high | No serious limitations | NA | –1: important inconsistency concerns | Inclusion of CP A patients may have confounded results | No uncertainty about directness | NA | –1: imprecise or sparse data | Small patient numbers (N=90) Potential for confounding due to open-label design | Moderate |
| Vogel et al 2020 [8]  KEYNOTE-240 | Poster | Randomised trial = high | –1: serious limitation to study quality | Retrospective analysis by ALBI grade, descriptive statistical analysis only | –1: important inconsistency concerns | ALBI grade 1 and 2 patients had different BCLC stage and AE types leading to sorafenib discontinuation | No uncertainty about directness | NA | –1: high probability of reporting bias | Retrospective analysis not blinded, and differences observed between treatment groups at baseline | Moderate |
| Vogel et al 2021 [25]  REFLECT | Full-text | Randomised trial = high | –1: serious limitation to study quality | Retrospective analysis of REFLECT trial stratifying patients by ALBI grade or CP, descriptive statistical analysis only | –1: important inconsistency concerns | Proportions of ALBI 1 and 2 patients differed between treatment groups | No uncertainty about directness | NA | –1: high probability of reporting bias | Potential for confounding due to differences in baseline characteristics between groups | Moderate |
| Vogel et al 2021 [9]  RESORCE | Poster | Randomised trial = high | –1: serious limitation to study quality | Retrospective analysis of RESORCE trial stratifying patients by ALBI grade | –1: important inconsistency concerns | Comparisons of ALBI 1 and 2 data are limited by the differences in baseline characteristics of these two populations | No uncertainty about directness | NA | –1: imprecise or sparse data | Excluding CP B and C patients limited the applicability of this study to those patients | Moderate |
| Yau et al 2009 [18] | Full-text | Non-randomised trial = moderate | –1: serious limitation to study quality | Single-arm study of sorafenib in patients with liver cirrhosis | –1: important inconsistency concerns | Almost three-quarters of patients are CP A with the remainder being CP B and C; 88% of patients are male | No uncertainty about directness | NA | –1: imprecise or sparse data | Small number of patients (N=51) | Moderate |
| Zhu et al 2013 [19] | Full-text | Non-randomised trial = moderate | –1: serious limitation to study quality | Single-arm study of ramucirumab, inclusion of patients with CP A and B, and also patients with liver transplant | –1: important inconsistency concerns | CP A population three times larger than CP B population | No uncertainty about directness | NA | –1: imprecise or sparse data | Small patient numbers (N=42). Subsequent use of sorafenib in progressing patients may have influenced PFS and TTP results | Moderate |
| Zhu et al 2017 [20]  REACH | Full-text | Randomised trial = high | –1: serious limitation to study quality | Retrospective analysis of REACH stratified treatment arms by CP score | –1: important inconsistency concerns | CP scores higher in groups with 5 and 6 than 7 and 8, which were combined (7+8) to create a group for analysis | No uncertainty about directness | NA | No modifying factors | NA | Moderate |

***Supplementary Table S4***. **GRADE quality appraisal and rating of each eligible publication**

**Interpretation:**

High = Further research is very unlikely to change our confidence in the estimate of effect.

Moderate = Further research is likely to have an important impact on our confidence in the estimate of effect and may change the estimate.

Low = Further research is very likely to have an important impact on our confidence in the estimate of effect and is likely to change the estimate.

Very low = Any estimate of effect is very uncertain.

*Modified ALBI grading was used in study (ALBI grade 2 was divided into ALBI 2a and 2b) [26].

ALBI=albumin–bilirubin grade. BCLC= Barcelona Clinic Liver Cancer. CP=Child–Pugh score. HBV=hepatitis B virus. HCC=hepatocellular carcinoma. HCV=hepatitis C virus. mALBI=modified albumin–bilirubin. NA=not applicable. PFS=progression-free survival. RCT=randomised controlled trial. TTP=time to progression.

| **First author Study name**  **Study identifier** | **Intervention (drug class) N** | **Liver function safety subgroups, n** | **Grade ≥3 AEs, %** | **Discontinuation rate, %** | **Dose reductions due to AEs, %** | **Evidence of association between liver function and safety profile** |
| --- | --- | --- | --- | --- | --- | --- |
| Kelley et al [5]  CELESTIAL  NCT01908426 | Cabozantinib (TKI); N=707 | **ALBI 1:** 102 **ALBI 2:** 133 | ALBI 1 vs ALBI 2  Any: 34% vs 38% PPE: 0% vs 0% Hypertension: 3% vs 1% AST increased: 4% vs 8% Fatigue: 2% vs 6% Diarrhoea: 1% vs 2% Asthenia: 2% vs 2% Decreased appetite: 0% vs 1% Anaemia: 1% vs 8% | ALBI 1 vs ALBI 2  2% vs 4% | NR | **Possibly**   - Possible trend towards lower rates of grade ≥3 AEs in the ALBI 1 vs ALBI 2 subgroup |
| Vogel et al [9] NCT01774344  RESORCE | Regorafenib (TKI); N=573 | **ALBI 1**: 81 **ALBI 2**: 111 | ALBI 1 vs ALBI 2  TEAEs Grade 3: 33% vs 31%  Grade 4: 2% vs 11% Grade 5: 16% vs 22% TRAEs Grade 3: 11% vs 20%  Grade 4: 1% vs 0% Grade 5: 0% vs 2% | ALBI 1 vs ALBI 2  11% vs 24%  Due to TRAE: ALBI 1 vs ALBI 2  1% vs 5% | ALBI 1 vs ALBI 2, due to:  TEAEs: 23% vs 36%  TRAEs: 6% vs 14% | **Possibly**   - ALBI 1 (vs ALBI 2) was associated with a lower rate of:   - some grade 3 TRAEs and TEAEs   - discontinuation due to TEAEs and TRAEs   - dose reductions due to TEAEs and TRAEs |
| Kudo et al [23] REACH & REACH-2 NCT01140347 NCT02435433 | Ramucirumab (mAb) N=857 | **ALBI 1**: 95 **ALBI 2**: 120 **ALBI 3**: 6 | Grade ≥3 AESIs  Liver injury/liver failure  ALBI 1: 18 (18·9%)  ALBI 2: 39 (32·5%)  ALBI 3: 2 (33·3%) | Discontinued because of AEs in at least two patients:  ALBI 1 vs ALBI 2  4·2% vs 12·5% | NR | **Possibly**   - ALBI 1 vs ALBI 2 was associated with lower rates of discontinuation |
| Brandi et al [21]*  REACH-2  NCT02435433 | Ramucirumab (mAb) N=292 | Ramucirumab + placebo **ALBI 1:** 143 **ALBI 2**: 144 | NR | NR | NR | **Yes**  ALBI 2 (vs ALBI 1) was associated with a higher proportion of grade ≥3 TEAEs |
| Zhu et al [20]  REACH NCT01140347 | Ramucirumab (mAb)  N=644 | **CP 5:** 180 **CP 6:** 100 **CP 7 or 8:** 39 | CP 5 vs CP 6 vs CP 7 or 8  41·2% vs 57·7% vs 63·2%  Grade ≥3 Peripheral oedema: 0·0% vs 1·0% vs 0·0% Fatigue: 2·3% vs 4·1% vs 0·0% Headache: 0·0% vs 0·0% vs 0·0%  Hypertension: 3·4% vs 4·1% vs 2·6% Decreased appetite: 1·1% vs 0·0% vs 0·0%  Abdominal pain: 2·8% vs 7·2% vs 2·6%  Ascites: 3·4% vs 4·1% vs 18·4%  Asthenia: 1·1% vs 3·1% vs 5·3% | NR | NR | **Yes**   - Patients with CP 7 or CP 8 (vs CP 6 or CP 5) had higher rates of:   - overall grade ≥3 TEAEs, including higher rates of hypertension, decreased appetite, ascites, asthenia |
| Brandi et al [21]*  REACH-2  NCT02435433 | Ramucirumab (mAb) N=292 | Ramucirumab + placebo **CP 5:** 177 **CP 6**: 115 | NR | NR | NR | **Yes**  CP 6 (vs CP 5) associated with a higher proportion of Grade ≥3 TEAEs |

***Supplementary Table S5.*** **Safety profile of placebo-treated patients by liver function subgroups, defined by ALBI grade or Child–Pugh score**

*Stratified results according to ALBI grade and by Child–Pugh score.

AE=adverse event. AESI=adverse event of special interest. ALBI=albumin–bilirubin grade. AST=aspartate aminotransferase. CP=Child–Pugh score. mAb=monoclonal antibody. NCT=National Clinical Trial. NR=not reported. PPE=palmar–plantar erythrodysesthesia. TEAE=treatment-emergent adverse event. TKI=tyrosine kinase inhibitor. TRAE=treatment-related adverse event.

**^
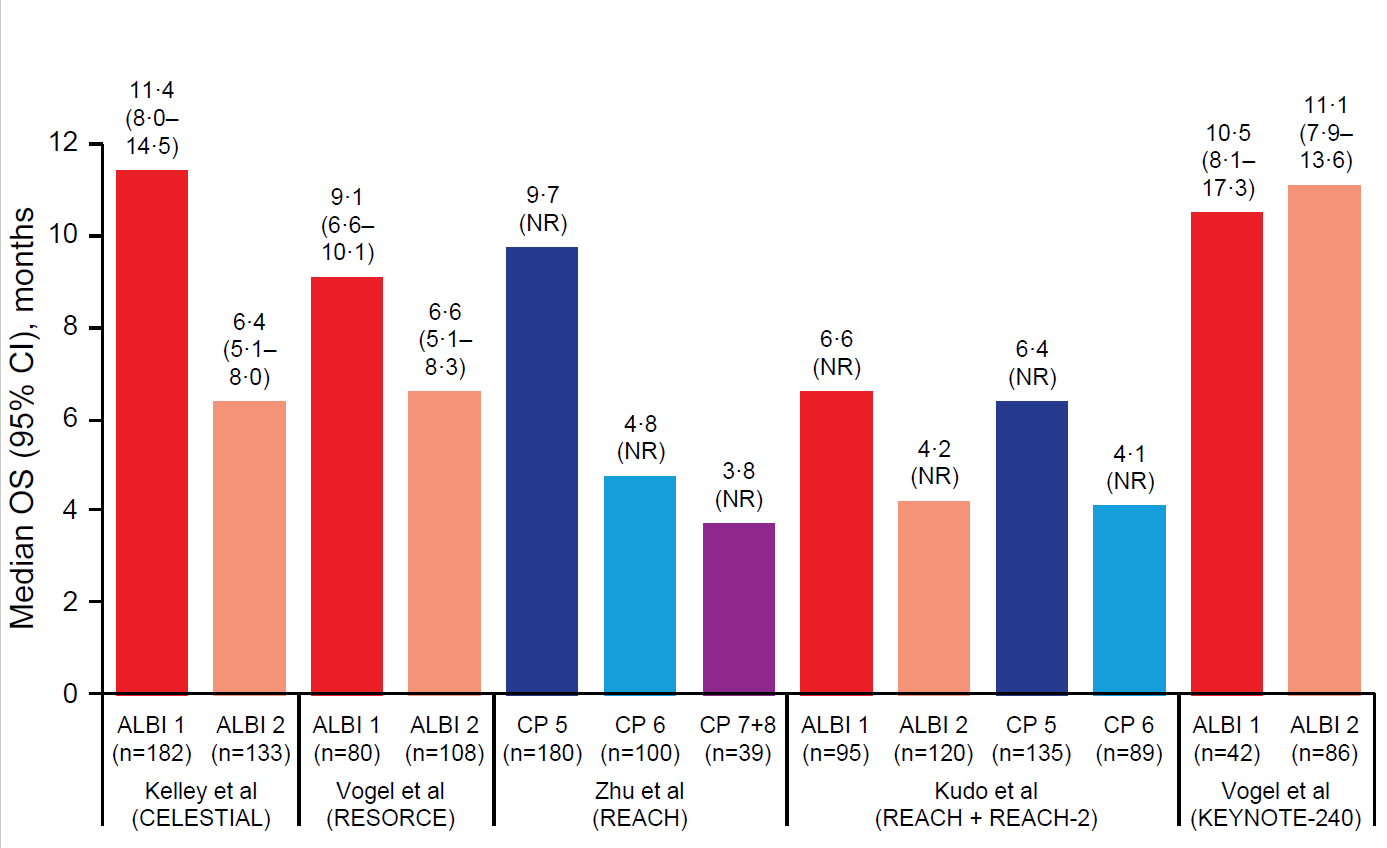
^**

***Supplementary Figure S1.* Prognostic data (placebo arm): median OS estimates; stratified by ALBI[5, 8, 9, 23]- and Child–Pugh[20, 23] -defined liver function subgroups***

*Kobayashi et al was not included in graph owing to small sample size (ALBI 1–2a, n=16; ALBI 2b, n=170), and no association between baseline liver function and OS was observed (ALBI 1–2a, 15.2 months; ALBI 2b, 15·1 months).

ALBI=albumin–bilirubin grade. CI=confidence interval. CP=Child–Pugh score. NR=not reported. OS=overall survival.


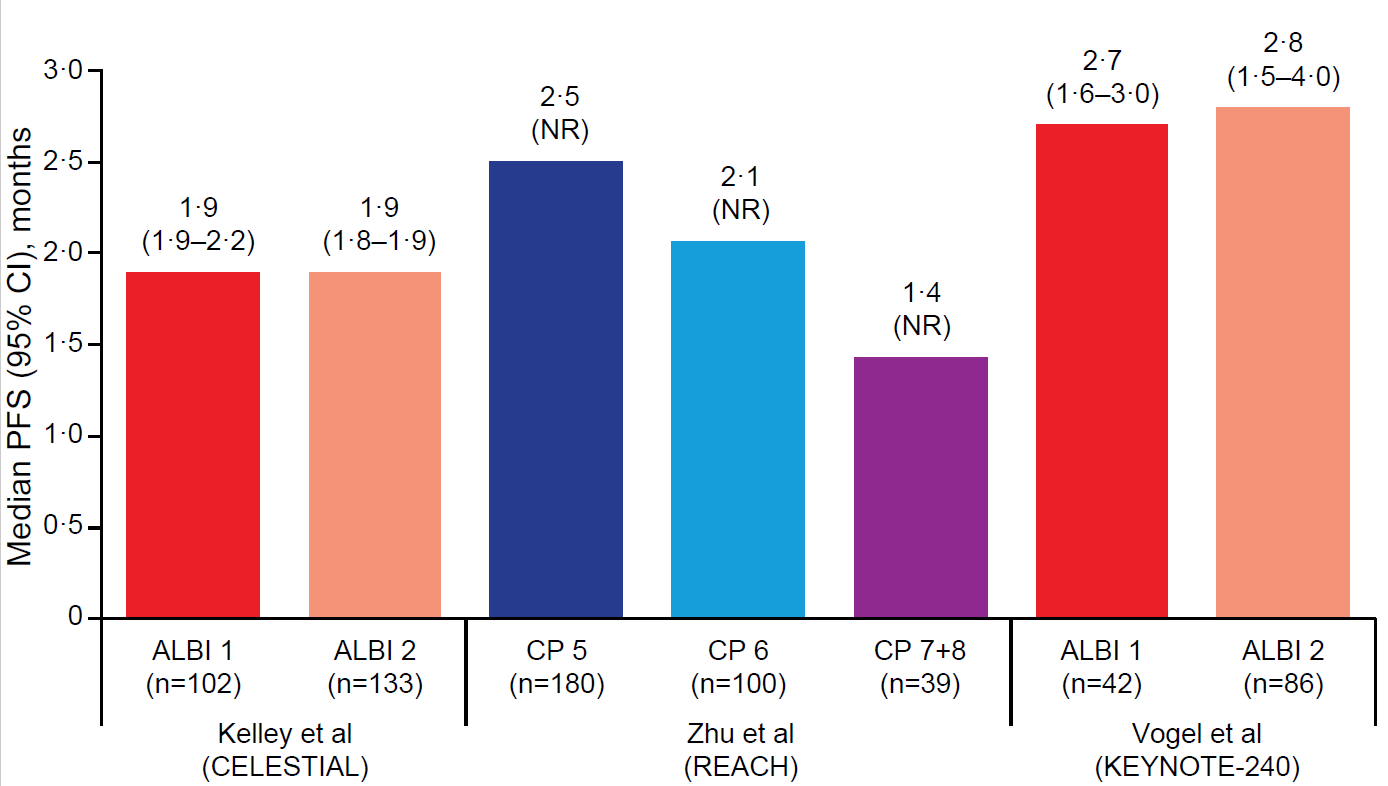


***Supplementary Figure S2.* Prognostic analysis (placebo arm): median PFS estimates, stratified by ALBI-defined liver function subgroups [5, 8, 20]**

ALBI=albumin–bilirubin. CI=confidence interval. CP=Child–Pugh score. NR=not reported. PFS=progression-free survival.

**
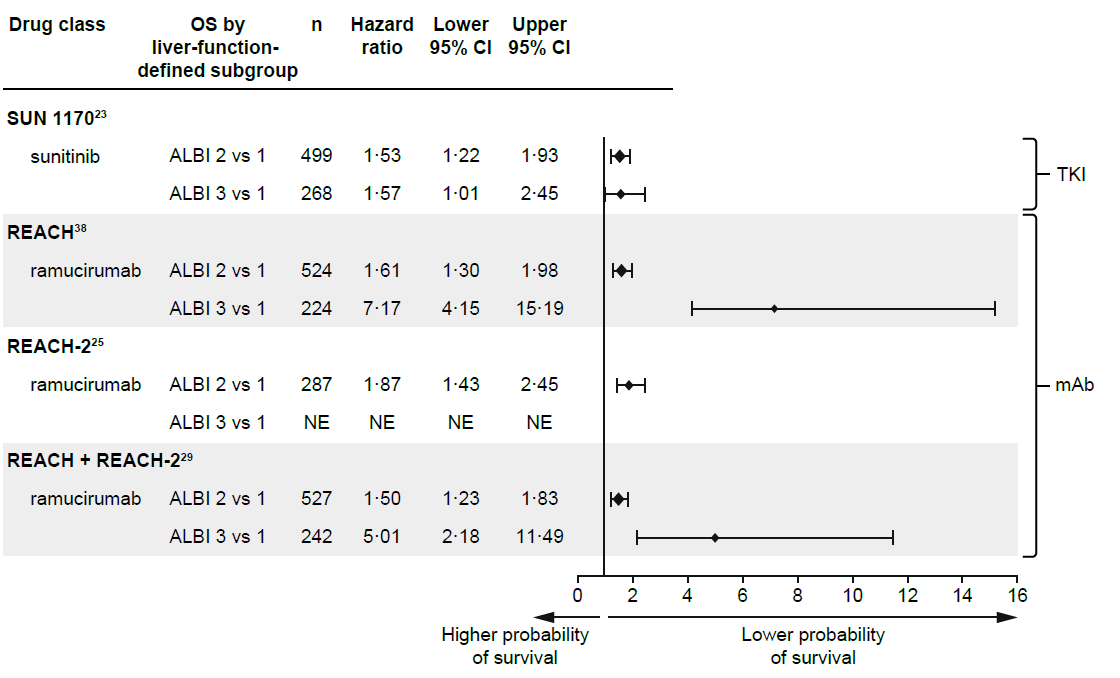
**

***Supplementary Figure S3.* Prognostic data: overall survival for within-active treatment group analyses, stratified by baseline ALBI grade[1, 23]**

ALBI=albumin–bilirubin. CI=confidence interval. mAb=monoclonal antibody. NE=not estimable. OS=overall survival. TKI=tyrosine kinase inhibitor.


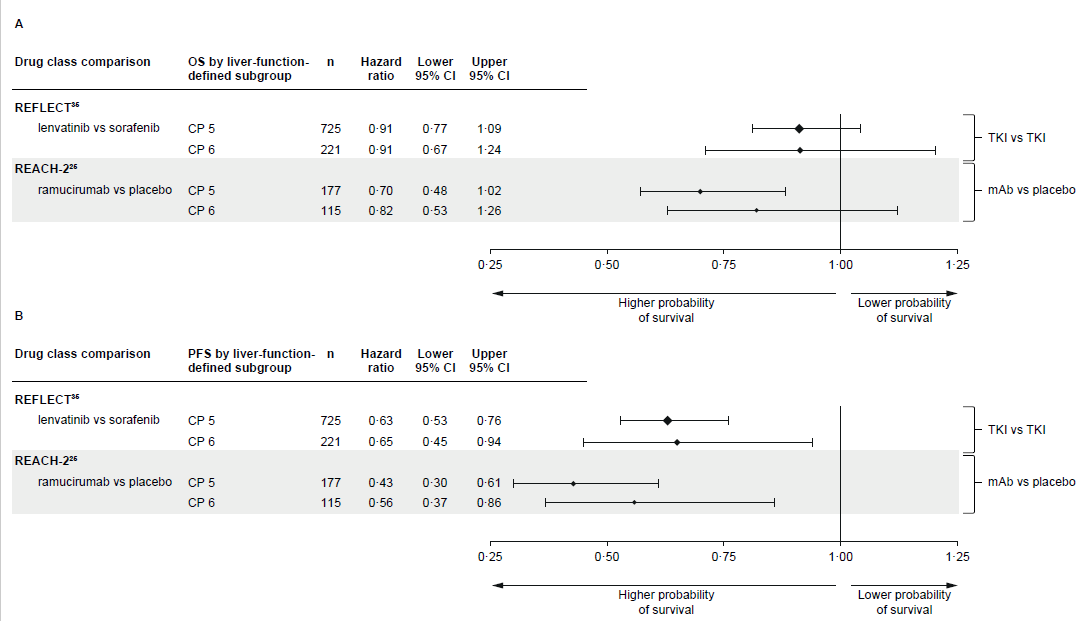


***Supplementary Figure S4.* Predictive analysis: forest plot for between-treatment-arm analyses (investigational drug vs comparator), stratified by liver function, PFS (A), OS (B)[21, 25]**
CI=confidence interval. CP=Child–Pugh. mAb=monoclonal antibody. OS=overall survival. PFS=progression-free survival. TKI=tyrosine kinase inhibitor.

**References**

[1] Abdel-Rahman O. Impact of baseline characteristics on outcomes of advanced HCC patients treated with sorafenib: a secondary analysis of a phase III study. J Cancer Res Clin Oncol 2018;144:901-908.

[2] Johnson PJ, Berhane S, Kagebayashi C, Satomura S, Teng M, Reeves HL, et al. Assessment of liver function in patients with hepatocellular carcinoma: a new evidence-based approach-the ALBI grade. J Clin Oncol 2015;33:550-558.

[3] Johnson PJ, Qin S, Park JW, Poon RT, Raoul JL, Philip PA, et al. Brivanib versus sorafenib as first-line therapy in patients with unresectable, advanced hepatocellular carcinoma: results from the randomized phase III BRISK-FL study. J Clin Oncol 2013;31:3517-3524.

[4] Cheng AL, Kang YK, Lin DY, Park JW, Kudo M, Qin S, et al. Sunitinib versus sorafenib in advanced hepatocellular cancer: results of a randomized phase III trial. J Clin Oncol 2013;31:4067-4075.

[5] Kelley RK, Miksad R, Cicin I, Chen Y, Klumpen HJ, Kim S, et al. Efficacy and safety of cabozantinib for patients with advanced hepatocellular carcinoma based on albumin-bilirubin grade. Br J Cancer 2021.

[6] Kobayashi S, Kondo M, Morimoto M, Hidaka H, Nakazawa T, Aikata H, et al. The influence of liver function on the outcomes of phase II trial of sorafenib vs. hepatic arterial infusion chemotherapy for advanced hepatocellular carcinoma (SO-6). Ann Oncol 2020;31.

[7] Kudo M, Finn RS, Cheng AL, Zhu AX, Ducreux M, Galle PR, et al. IMbrave150: Albumin-Bilirubin Grade Analyses in a Phase III Study of Atezolizumab + Bevacizumab vs Sorafenib in Patients With Unresectable Hepatocellular Carcinoma (O-18). ILCA Annual Conference 2021.

[8] Vogel A, Merle P, Verslype C, Finn RS, Zhu AX, Cheng AL, et al. Baseline liver function and outcomes in patients with unresectable hepatocellular carcinoma in KEYNOTE-240 (1003P). Ann Oncol 2020;31:S696-S697.

[9] Vogel A, Merle P, Granito A, Ikeda M, LeBerre MA, Ozgurdal K, et al. Outcomes based on albumin-bilirubin grade in the phase 3 RESORCE trial of regorafenib versus placebo in patients with advanced hepatocellular carcinoma (P026). European Association for the Study of the Digital Liver Cancer Summit 2021.

[10] Bruix J, Qin S, Merle P, Granito A, Huang YH, Bodoky G, et al. Regorafenib for patients with hepatocellular carcinoma who progressed on sorafenib treatment (RESORCE): a randomised, double-blind, placebo-controlled, phase 3 trial. Lancet 2017;389:56-66.

[11] Abou-Alfa GK, Amadori D, Santoro A, Figer A, De Greve J, Lathia C, et al. Safety and Efficacy of Sorafenib in Patients with Hepatocellular Carcinoma (HCC) and Child-Pugh A versus B Cirrhosis. Gastrointest Cancer Res 2011;4:40-44.

[12] El-Khoueiry A, Meyer T, Cheng A, Rimassa L, Sen S, Milwee S, et al. Outcomes for patients with advanced hepatocellular carcinoma and Child-Pugh B liver function in the phase 3 CELESTIAL study of cabozantinib vs placebo (SO-9). Ann Oncol 2020;31:S220.

[13] Huynh J, Cho MT, Kim EJH, Ren M, Robbins C, Amaya-Chanaga C, et al. Post hoc analysis in patients with unresectable hepatocellularcarcinoma who progressed to Child-Pugh B liver function in the phase 3 REFLECT study of lenvatinib (poster P035). Journal of Clinical Oncology (Poster presented at the: Canadian Liver Meeting (CLM) 2021; May 2–5, 2021) 2021;39:298-298.

[14] Kudo M, Finn RS, Qin S, Han KH, Ikeda K, Piscaglia F, et al. Lenvatinib versus sorafenib in first-line treatment of patients with unresectable hepatocellular carcinoma: a randomised phase 3 non-inferiority trial. Lancet 2018;391:1163-1173.

[15] Pressiani T, Boni C, Rimassa L, Labianca R, Fagiuoli S, Salvagni S, et al. Sorafenib in patients with Child-Pugh class A and B advanced hepatocellular carcinoma: a prospective feasibility analysis. Ann Oncol 2013;24:406-411.

[16] Suzuki E, Kaneko S, Okusaka T, Ikeda M, Yamaguchi K, Sugimoto R, et al. A multicenter Phase II study of sorafenib in Japanese patients with advanced hepatocellular carcinoma and Child Pugh A and B class. Jpn J Clin Oncol 2018;48:317-321.

[17] Thomas MB, Garrett-Mayer E, Anis M, Anderton K, Bentz T, Edwards A, et al. A Randomized Phase II Open-Label Multi-Institution Study of the Combination of Bevacizumab and Erlotinib Compared to Sorafenib in the First-Line Treatment of Patients with Advanced Hepatocellular Carcinoma. Oncology 2018;94:329-339.

[18] Yau T, Chan P, Ng KK, Chok SH, Cheung TT, Fan ST, et al. Phase 2 open-label study of single-agent sorafenib in treating advanced hepatocellular carcinoma in a hepatitis B-endemic Asian population: presence of lung metastasis predicts poor response. Cancer 2009;115:428-436.

[19] Zhu AX, Finn RS, Mulcahy M, Gurtler J, Sun W, Schwartz JD, et al. A phase II and biomarker study of ramucirumab, a human monoclonal antibody targeting the VEGF receptor-2, as first-line monotherapy in patients with advanced hepatocellular cancer. Clin Cancer Res 2013;19:6614-6623.

[20] Zhu AX, Baron AD, Malfertheiner P, Kudo M, Kawazoe S, Pezet D, et al. Ramucirumab as Second-Line Treatment in Patients With Advanced Hepatocellular Carcinoma: Analysis of REACH Trial Results by Child-Pugh Score. JAMA Oncol 2017;3:235-243.

[21] Brandi G, Kudo M, Kang YK, Yen CJ, Finn R, Galle P, et al. Ramucirumab for patients with hepatocellular carcinoma and elevated alpha-fetoprotein following sorafenib treatment: exploratory analysis of REACH-2 trial results by albumin-bilirubin grade and Child-Pugh score (OP-07). HCC Summit 2019 2019:35-36.

[22] Zhu AX, Kang YK, Yen CJ, Finn RS, Galle PR, Llovet JM, et al. Ramucirumab after sorafenib in patients with advanced hepatocellular carcinoma and increased alpha-fetoprotein concentrations (REACH-2): a randomised, double-blind, placebo-controlled, phase 3 trial. Lancet Oncol 2019;20:282-296.

[23] Kudo M, Galle PR, Brandi G, Kang YK, Yen CJ, Finn RS, et al. Effect of ramucirumab on ALBI grade in patients with advanced HCC: Results from REACH and REACH-2. JHEP Rep 2021;3:100215.

[24] Kudo M, Matilla A, Santoro A, Melero I, Gracian AC, Acosta-Rivera M, et al. CheckMate 040 Cohort 5: A phase I/II study of nivolumab in patients with advanced hepatocellular carcinoma and Child-Pugh B cirrhosis. J Hepatol 2021;75:600–609.

[25] Vogel A, Frenette C, Sung M, Daniele B, Baron A, Chan SL, et al. Baseline Liver Function and Subsequent Outcomes in the Phase 3 REFLECT Study of Patients with Unresectable Hepatocellular Carcinoma. Liver Cancer 2021;10:510–521.

[26] Hiraoka A, Michitaka K, Kumada T, Izumi N, Kadoya M, Kokudo N, et al. Validation and Potential of Albumin-Bilirubin Grade and Prognostication in a Nationwide Survey of 46,681 Hepatocellular Carcinoma Patients in Japan: The Need for a More Detailed Evaluation of Hepatic Function. Liver Cancer 2017;6:325-336.
